# Cross-Species Transcriptomic Analysis Identifies an Endocannabinoid-Associated Immune Remodeling Signature and Candidate Pharmacologic Targets in Spinal Cord Injury

**DOI:** 10.3390/biomedicines14071446

**Published:** 2026-06-25

**Authors:** Tamer Tamdogan, Sevim Ondul, Muharrem Furkan Yuzbasi, Ibrahim Yilmaz

**Affiliations:** 1 Department of Neurosurgery, Giresun University Faculty of Medicine, Giresun 28200, Türkiye; 2 Department of Neurosurgery, Kahramanmaras Sutcu Imam University Faculty of Medicine, Kahramanmaras 46040, Türkiye; 3 Unit of Pharmacovigilance, Dr. Ismail Fehmi Cumalioglu City Hospital, Ministry of Health of the Republic of Türkiye, Tekirdag 59020, Türkiye

**Keywords:** *CNR2*, drug–gene interaction, endocannabinoid system, immune remodeling, *PTGS2*, spinal cord injury, transcriptomics

## Abstract

**Background**: Spinal cord injury (SCI) triggers secondary injury processes involving neuroinflammation and systemic immune remodeling. The endocannabinoid system (ECS) has been implicated in neuroimmune regulation, but its transcriptional relationship with immune remodeling and its translational relevance in human SCI blood remain incompletely defined. **Methods**: A cross-species discovery–validation–translation framework was applied using a rat spinal cord discovery cohort (GSE45006), an independent mouse validation cohort (GSE171441), and a human peripheral white blood cell cohort (GSE151371). Analyses included differential expression profiling, ECS-focused assessment, cross-species comparison, immune-cell signature scoring, ECS–immune correlation analysis, receiver operating characteristic (ROC) analysis, LASSO-based biomarker prioritization, network analysis, disease enrichment, drug–gene interaction querying, and transcription factor/microRNA regulatory annotation. **Results**: ECS-related transcriptional remodeling was identified across rodent and human datasets in a compartment-dependent manner. In human SCI blood, *CNR2*, *PTGS2*, and *DAGLB* were significantly altered and showed biomarker-prioritization potential. Human SCI blood also showed innate immune enrichment, adaptive immune depletion, and significant ECS–immune correlations. The integrated 28-gene SCI–ECS immune panel formed a functionally coherent protein–protein interaction (PPI) network enriched in immune-response pathways. Disease enrichment supported an immune/inflammatory pathological context, whereas DGIdb identified hypothesis-generating drug–gene relationships involving ECS-related targets. ChEA 2022 revealed nominal transcription factor annotations that did not survive multiple-testing correction, and miRNet identified database-derived miRNA regulators of panel genes. In a secondary sensitivity analysis, the combined ECS signature also retained discriminatory performance against non-CNS trauma controls, suggesting that the observed transcriptional pattern was not fully attributable to generalized trauma-related responses. **Conclusions**: This study proposes an ECS-associated immune remodeling signature in SCI with translational biomarker-prioritization and pharmacologic target-annotation context in human peripheral blood. These findings are exploratory and require prospective and functional validation.

## 1. Introduction

Traumatic spinal cord injury (SCI) remains one of the most consequential forms of neurotrauma, frequently leaving survivors with permanent motor, sensory, and autonomic impairments and a long horizon of secondary complications. Beyond the immediate mechanical insult, the evolving secondary injury cascade creates a moving therapeutic target—one that is shaped by time, injury heterogeneity, and tissue compartment-specific biology. These realities continue to complicate preclinical-to-clinical translation, where apparently plausible interventions may falter when confronted with differences in injury dynamics, outcome measures, and patient variability. Accordingly, there is a sustained need for molecularly informed frameworks that can better align mechanistic understanding with biomarker development and therapeutic prioritization in SCI [1,2].

A central feature of the post-SCI cascade is neuroinflammation, which encompasses both local and systemic immune remodeling. Innate immune activation and myeloid recruitment may coexist with qualitative and quantitative shifts in adaptive immune states, and the net balance can vary across injury stage and biological compartment. Importantly, peripheral blood provides a clinically accessible window into systemic immune alterations, and transcriptomic profiling—particularly when anchored by cell-resolved or immune-state-aware analyses—may help define immune signatures that support patient stratification and biomarker development. Recent work applying single-cell transcriptomics to circulating leukocytes in SCI-relevant experimental settings underscores both the complexity and the translational promise of blood-based immune readouts, while contemporary reviews continue to refine the interpretive context of inflammatory signaling and cellular infiltration across stages of SCI [3,4].

Within this landscape, the endocannabinoid system (ECS) offers a biologically coherent rationale for investigation at the intersection of neuroimmune regulation and lipid signaling. *CNR2* (CB2) is widely discussed as an immunomodulatory receptor with relevance to activated immune and glial populations, providing a plausible link between immune remodeling and injury-context signaling without presuming a single dominant mechanism. *PTGS2* (COX-2), beyond its canonical prostanoid biology, sits at a lipid-inflammatory interface that can connect prostanoid production with endocannabinoid-related substrates and signaling balance; experimental work in spinal glial systems supports the concept of an endocannabinoid–prostanoid switch under reactive conditions, which may be relevant when considering inflammatory remodeling after central nervous system (CNS) injury. Finally, *DAGLB*, as a diacylglycerol lipase implicated in 2-arachidonoylglycerol (2-AG) biosynthesis, supports the biochemical rationale for examining *DAGLB* within an ECS-focused transcriptomic panel and for interpreting ECS-associated transcriptional patterns in the broader context of lipid metabolism [5,6].

Despite growing interest in both immune signatures and lipid signaling in SCI, it remains unclear how ECS transcriptional remodeling relates to immune remodeling, whether such relationships show cross-species reproducibility, and whether they can yield a clinically meaningful signature in human blood for biomarker and drug-repurposing applications.

This study tested the hypothesis that SCI-associated ECS transcriptional alterations are linked to immune remodeling, show partial cross-species conservation, and support a translational ECS–immune signature with biomarker and drug-repurposing relevance in human SCI blood. A discovery–validation–translation framework was applied using a rat spinal cord discovery cohort (GSE45006), an independent mouse spinal cord validation cohort (GSE171441), and a human peripheral white blood cell translational cohort (GSE151371). The analytical design was interpreted within the limits of transcriptomic and network-based translational bioinformatics, without implying causal mechanisms or therapeutic efficacy [1,2].

## 2. Materials and Methods

### 2.1. Study Design

This study was designed as a cross-species transcriptomic analysis to identify endocannabinoid-system (ECS)-associated molecular alterations after SCI, validate their reproducibility across species, and determine their relationship with immune remodeling and biomarker performance. The analytical strategy consisted of three sequential layers: a rat discovery cohort, an independent mouse validation cohort, and a human translational cohort.

The rat dataset GSE45006 was selected as the discovery cohort because it represents a genome-wide transcriptomic analysis of thoracic spinal cord tissue after aneurysm clip impact-compression SCI, a model designed to reproduce key mechanical features of human traumatic SCI, including acute impact and persistent compression [7]. This cohort was used to identify temporal SCI-associated transcriptional alterations and to define the initial ECS remodeling profile. The mouse dataset GSE171441 was used as an independent biological validation cohort. This dataset contains RNA-sequencing profiles from injured mouse spinal cord tissue after SCI and was used to assess whether ECS-related transcriptional changes identified in the rat discovery analysis were reproducible in a second experimental species [8]. The human dataset GSE151371 was used as the translational cohort. This dataset contains peripheral white blood cell RNA-sequencing profiles from healthy controls, non-CNS trauma controls, and individuals with traumatic SCI during the acute injury phase [9].

In the present study, healthy controls and SCI patients were analyzed to evaluate human ECS gene alterations, immune-cell signature-score remodeling, ECS–immune correlations, and the diagnostic performance of ECS-derived biomarkers.

**Figure 1:**
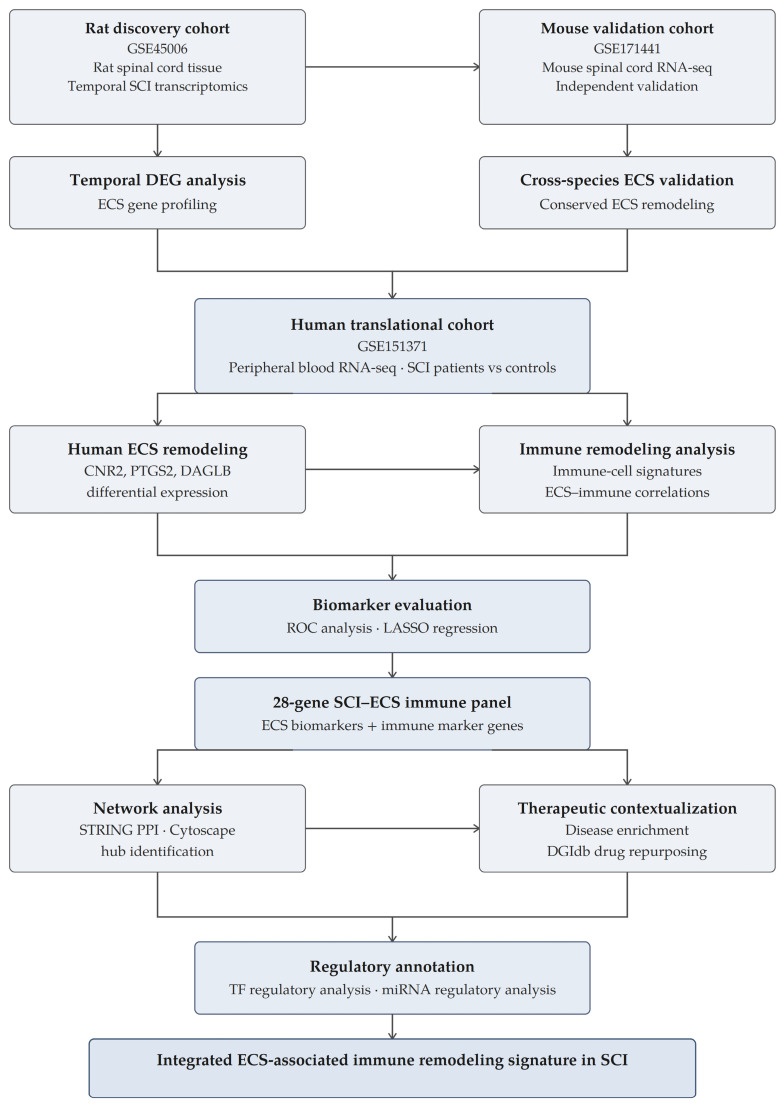
Study design and analytical workflow. The study integrated three public transcriptomic datasets within a discovery–validation–translation framework. GSE45006 was used as the rat SCI discovery cohort for temporal differential expression analysis and ECS gene profiling, whereas GSE171441 served as an independent mouse validation cohort for cross-species assessment of ECS-associated transcriptional remodeling. GSE151371 was used as the human peripheral blood translational cohort to evaluate ECS gene alterations, immune-cell signature scores, ECS–immune correlations, and biomarker performance using ROC and LASSO analyses. ECS-related biomarker genes and immune marker genes were then integrated into a 28-gene SCI–ECS immune panel for STRING PPI analysis, Cytoscape hub identification, disease enrichment, DGIdb drug repurposing, and transcription factor (TF)/microRNA (miRNA) regulatory annotation.

The study design and analytical workflow are summarized in Figure 1.

### 2.2. Public Transcriptomic Datasets

Three publicly available transcriptomic datasets were retrieved from the National Center for Biotechnology Information (NCBI) Gene Expression Omnibus (GEO) database and analyzed according to the study workflow described in Figure 1. Dataset selection was based on three predefined criteria: (i) availability of SCI-related transcriptomic profiles, (ii) inclusion of appropriate control groups, and (iii) suitability for cross-species validation of ECS-associated molecular alterations.

The discovery cohort consisted of GSE45006, a rat spinal cord transcriptomic dataset originally reported by Chamankhah et al. [7] and deposited in the Gene Expression Omnibus [10]. This dataset was generated using an aneurysm clip impact-compression SCI model, which reproduces key pathological characteristics of human traumatic SCI, including acute mechanical impact followed by sustained compression. Thoracic spinal cord tissue was collected from the lesion epicenter and profiled using the Affymetrix Rat Genome 230 2.0 Array platform (GPL1355). The dataset comprises samples representing acute, subacute, and chronic phases after injury, enabling evaluation of temporal transcriptional remodeling following SCI.

The biological validation cohort consisted of GSE171441, an independent mouse SCI RNA-sequencing dataset reported by Patel et al. [8] and publicly available through the Gene Expression Omnibus [11]. In the original study, spinal cord tissue surrounding the lesion site was collected at 3, 14, and 35 days post-injury and subjected to transcriptomic profiling using the Illumina MiSeq platform (GPL16417). To maximize comparability with the temporal framework established in the rat discovery cohort, analyses in the present study focused on the 3-day and 35-day post-injury time points, representing acute and chronic stages of SCI, respectively. This cohort was used to determine whether ECS-associated transcriptional alterations identified in rats were reproducible in an independent experimental species.

The translational cohort consisted of GSE151371, a human peripheral blood RNA-sequencing dataset reported by Kyritsis et al. [9] and deposited in the Gene Expression Omnibus [12]. This dataset contains 58 sequenced samples, including 10 healthy controls, 10 non-CNS trauma controls, and 38 patients with acute traumatic SCI. Gene-expression profiling was performed using the Illumina HiSeq 4000 platform (GPL20301). Because the primary objective of the present study was to evaluate SCI-associated molecular alterations, downstream analyses were restricted to healthy controls and SCI patients. This cohort was used to determine whether ECS-related transcriptional signatures identified in experimental SCI models could be detected in human peripheral blood and to investigate their association with immune remodeling and biomarker performance.

The three datasets collectively provided a discovery–validation–translation framework spanning experimental rodent SCI models and human clinical SCI.

### 2.3. Differential Expression Analysis

Differential expression analyses were performed independently within each dataset, taking into account platform type, data structure, and experimental design.

For the rat discovery cohort (GSE45006), processed Affymetrix microarray expression data were retrieved from GEO and analyzed at each post-injury time point against sham controls. Linear modeling was performed using the limma framework, and moderated statistics were obtained by empirical Bayes moderation. Probe-level results were annotated to gene symbols using the corresponding Affymetrix Rat Genome 230 2.0 Array annotation platform (GPL1355). When multiple probes mapped to the same gene symbol, gene-level results were retained according to adjusted *p* value and absolute log fold-change (logFC) to generate non-redundant gene-level differential expression tables [7,13].

For the mouse validation cohort (GSE171441), processed HTSeq count data were downloaded from GEO. Untreated SCI-control and sham samples were retained for analysis at 3 and 35 days post-injury. Count data were analyzed using an edgeR–voom–limma workflow. Lowly expressed genes were filtered using ‘filterByExpr’, library sizes were normalized using trimmed mean of M values normalization, mean–variance modeling was performed using ‘voom’, and differential expression was estimated using limma linear modeling followed by empirical Bayes moderation. SCI samples were compared with time-matched sham samples at 3 and 35 days post-injury [8,13,14,15].

For the human translational cohort (GSE151371), the normalized ComBat-corrected expression matrix was downloaded from GEO. This processed matrix was selected because it had been normalized and batch-corrected by the original investigators, providing a harmonized expression resource suitable for downstream ECS-focused differential expression, immune-signature scoring, correlation, ROC, and LASSO analyses. Differential expression analysis was performed using limma by comparing SCI patients with healthy controls. Non-CNS trauma controls were not included in the primary SCI-versus-healthy-control contrast. As a secondary sensitivity analysis requested during peer review, differential expression analysis was additionally performed by comparing SCI patients with non-CNS trauma controls using the same limma framework. Gene-level statistics included logFC, average expression, moderated t statistic, nominal *p* value, Benjamini–Hochberg adjusted *p* value, and B statistic [9,13,16].

Across all cohorts, genes with an adjusted *p* value < 0.05 were considered statistically significant. For genome-wide differentially expressed gene (DEG) summary tables, an additional absolute log fold-change (logFC) threshold > 1 was applied when counting upregulated and downregulated DEGs.

### 2.4. ECS Gene Selection and Cross-Species ECS Profiling

A focused ECS gene panel was established prior to downstream analyses based on the biological roles of ECS components in cannabinoid signaling, endocannabinoid synthesis, endocannabinoid degradation, and lipid-mediated inflammatory regulation. The core ECS panel included cannabinoid receptors (*CNR1*/*Cnr1* and *CNR2*/*Cnr2*), endocannabinoid-degrading enzymes (*FAAH*/*Faah*, *MGLL*/*Mgll*, and *NAAA*/*Naaa*), diacylglycerol lipases involved in endocannabinoid biosynthesis (DAGLA/Dagla and *DAGLB*/*Daglb*), the serine hydrolase *ABHD6*/*Abhd6*, and the inflammation-associated lipid-signaling enzyme *PTGS2*/*Ptgs2*. The ECS panel was intentionally restricted to a focused set of canonical and functionally proximal ECS-related genes rather than designed as a comprehensive lipid-signaling or eicosanoid-pathway panel. *CNR1* and *CNR2* were included as the principal cannabinoid receptors; *FAAH*, *MGLL*, *NAAA*, and *ABHD6* were included as major endocannabinoid hydrolytic enzymes; *DAGLA* and *DAGLB* were included because of their role in 2-arachidonoylglycerol biosynthesis; and *PTGS2* was included because of its established position at the prostanoid–endocannabinoid inflammatory interface. Genes such as *ABHD12*, *GDE1*, *PLA2*-family enzymes, *TRPV1*, *PPARA*/*PPARG*, and broader eicosanoid-pathway components were not included in the predefined primary ECS panel because the objective was to test a compact ECS-focused transcriptomic hypothesis across heterogeneous platforms and species, rather than to perform an exhaustive lipid-signaling pathway screen.

Following genome-wide differential expression analysis, ECS genes were extracted from each dataset and evaluated separately in rat, mouse, and human cohorts. For each ECS gene, logFC values, nominal *p* values, and adjusted *p* values were recorded. Heatmaps and expression matrices were generated to visualize temporal and species-specific transcriptional patterns.

Cross-species integration was subsequently performed by comparing the direction, magnitude, and statistical significance of ECS-associated transcriptional alterations across the three cohorts. Particular emphasis was placed on characterizing cross-species and cross-compartment ECS remodeling patterns across independent experimental species and within the human translational cohort.

To facilitate biological interpretation, ECS genes were categorized according to their predominant expression pattern following SCI, including persistent downregulation, persistent upregulation, biphasic regulation, and time-dependent remodeling. These analyses formed the basis for defining ECS-associated remodeling patterns after SCI while accounting for species- and compartment-dependent differences.

### 2.5. Human Immune Remodeling Analysis

To investigate peripheral immune remodeling after SCI, immune-cell signature scores were estimated from the human transcriptomic dataset (GSE151371). Because the primary objective was to characterize broad immune-cell population shifts associated with SCI rather than to perform full deconvolution of bulk RNA-sequencing data, a marker-based immune scoring strategy was employed. Accordingly, the resulting scores were interpreted as relative lineage-associated expression signatures rather than absolute immune-cell fractions. Full immune deconvolution methods such as xCell or CIBERSORT were not applied, because the analysis was designed to assess broad directional immune remodeling using predefined marker sets rather than to infer detailed immune-cell proportions from bulk RNA-sequencing data.

Representative marker genes were selected for neutrophils, monocytes, macrophages, T cells, CD8 T cells, B cells, and natural killer (NK) cells based on established lineage-associated transcripts. For each immune-cell population, a signature score was calculated as the mean normalized expression of all available marker genes within the corresponding marker set. Immune-cell scores were generated independently for each sample.

To quantify SCI-associated immune remodeling, immune-cell signature scores were compared between SCI patients and healthy controls. Mean signature scores were calculated for each group, and differences were expressed as the mean score difference between SCI and healthy control samples. Statistical significance was evaluated using two-sided Student’s *t*-tests, and multiple-testing correction was performed using the Benjamini–Hochberg false-discovery-rate procedure [16].

Immune-cell populations were subsequently classified according to the direction of change after SCI, allowing identification of innate immune populations enriched in SCI and adaptive immune populations reduced after injury. The resulting immune-remodeling profile was visualized using bar-plot representations and used for subsequent ECS–immune interaction analyses.

### 2.6. ECS–Immune Correlation Analysis

To investigate the relationship between ECS dysregulation and peripheral immune remodeling after SCI, correlation analyses were performed between ECS gene expression levels and immune-cell signature scores in the human translational cohort (GSE151371).

Normalized expression values for the predefined ECS gene panel (*CNR1*, *CNR2*, *FAAH*, *MGLL*, *NAAA*, *PTGS2*, *DAGLA*, *DAGLB*, and *ABHD6*) were extracted from the human transcriptomic dataset. Immune-cell signature scores generated as described above were subsequently matched to the corresponding samples. Associations between ECS gene expression levels and immune-cell signature scores were evaluated using Spearman rank correlation analysis. Spearman’s method was selected because it does not assume a normal distribution and is robust for detecting monotonic relationships between biological variables. Correlation coefficients (ρ) and corresponding *p* values were calculated for every ECS gene–immune cell pair.

To account for multiple comparisons, *p* values were adjusted using the Benjamini–Hochberg false-discovery-rate procedure [16]. Correlation results were ranked according to adjusted *p* values, and statistically significant associations were interpreted as evidence of coordinated ECS–immune remodeling following SCI. For visualization, correlation coefficients were assembled into a gene-by-cell-type matrix and displayed as clustered heatmaps. Hierarchical clustering was applied to both ECS genes and immune-cell populations to identify groups exhibiting similar correlation patterns. Significant correlations were annotated within the heatmap to facilitate interpretation of ECS-associated immune signatures.

### 2.7. Receiver Operating Characteristic (ROC) Analysis

ROC analysis was performed to evaluate the ability of ECS-associated genes to discriminate between SCI patients and healthy controls in the human translational cohort (GSE151371). Only healthy control and SCI samples were included in ROC analyses, whereas non-CNS trauma controls were excluded from the primary diagnostic comparison. Based on differential expression results, *CNR2*, *PTGS2*, and *DAGLB* were selected for biomarker evaluation because these genes demonstrated significant SCI-associated transcriptional alterations within the human cohort. ROC curves were generated individually for each gene using normalized expression values as predictor variables and disease status (SCI versus healthy control) as the outcome variable.

To evaluate the potential benefit of a multigene ECS signature, a logistic regression model incorporating *CNR2*, *PTGS2*, and *DAGLB* was constructed. Predicted probabilities generated by the model were subsequently used to generate a combined ROC curve representing the diagnostic performance of the three-gene ECS panel. Diagnostic accuracy was quantified using the area under the curve (AUC). An AUC value of 0.5 was considered indicative of no discriminatory ability, whereas increasing AUC values reflected progressively improved classification performance. ROC analyses and AUC calculations were performed using the pROC package in R [17].

As a secondary analysis requested during peer review, ROC analyses were additionally performed using SCI patients versus non-CNS trauma controls. *CNR2*, *PTGS2*, and *DAGLB* were evaluated individually, and a combined three-gene logistic regression model was constructed to assess whether the ECS-associated signature retained discriminatory performance beyond general trauma-related responses.

### 2.8. LASSO-Based Machine-Learning Signature Construction

To identify a parsimonious ECS-based diagnostic signature, least absolute shrinkage and selection operator (LASSO) logistic regression was performed using the glmnet package in R. The analysis was restricted to ECS-related genes evaluated in the human translational cohort (GSE151371), including *CNR1*, *CNR2*, *FAAH*, *MGLL*, *NAAA*, *PTGS2*, *DAGLA*, *DAGLB*, and *ABHD6*. SCI samples and healthy controls were included in the machine-learning analysis, whereas trauma controls were excluded. Disease status (SCI versus healthy control) was used as the binary outcome variable. Gene expression values were standardized before model fitting, and LASSO regularization was applied using α = 1. Given the limited sample size, leave-one-out cross-validation (LOOCV) was used to determine the optimal regularization parameter (λ). The λ1se criterion was selected for model construction. Genes with non-zero coefficients at λ1se were retained in the final ECS signature. A composite LASSO score was calculated as the weighted sum of selected gene-expression values and their corresponding regression coefficients. The discriminatory performance of the final ECS signature was evaluated using ROC analysis and area under the curve (AUC) estimation. As a secondary sensitivity analysis requested during peer review, LASSO modeling was additionally performed using SCI patients and non-CNS trauma controls to assess whether the ECS signature remained informative beyond generalized trauma-associated transcriptional responses.

### 2.9. PPI Network Analysis

PPI network analysis was performed using the STRING database (version 12.0; https://string-db.org/; accessed on 8 June 2026) [18]. A total of 28 genes, comprising the three ECS-related biomarker genes identified through differential expression, ROC, and least absolute shrinkage and selection operator (LASSO) analyses (*CNR2*, *PTGS2*, and *DAGLB*), together with 25 immune-cell marker genes used for immune-remodeling assessment, were submitted to STRING for network construction. The analysis was restricted to *Homo sapiens.* To improve network reliability, a high-confidence interaction score threshold of 0.700 was applied. All available evidence channels implemented within STRING, including experimentally validated interactions, curated database annotations, co-expression, gene fusion, co-occurrence, neighborhood associations, and text-mining evidence, were retained. No additional first-shell or second-shell interactors were introduced, and the network was constructed exclusively from the uploaded query proteins. Functional enrichment analyses were performed within the STRING platform using Gene Ontology biological process (GO-BP), molecular function (GO-MF), and cellular component (GO-CC) annotations, together with Kyoto Encyclopedia of Genes and Genomes (KEGG) and Reactome pathway databases. Enrichment significance was assessed using false discovery rate (FDR)-adjusted *p* values as implemented in STRING. The resulting PPI network and enrichment profiles were used to characterize the biological organization, molecular functions, cellular localization patterns, and signaling pathways associated with ECS-linked immune remodeling following SCI. Because the present study was based on transcriptomic datasets, network analyses were interpreted as systems-level functional contextualization rather than evidence of direct causal or mechanistic relationships.

### 2.10. Hub Gene Identification and Network Topology Analysis

PPI network generated from the ECS–immune intersecting genes was imported into Cytoscape (version 3.10.4; https://cytoscape.org/; accessed on 8 June 2026) [19] for network visualization and topological analysis. Degree, betweenness centrality, and closeness centrality values were calculated using the Analyze Network function. Hub genes were identified using the maximal clique centrality (MCC) algorithm implemented in the cytoHubba plugin (version 0.1; available online: http://apps.cytoscape.org/apps/cytohubba; accessed on 8 June 2026). Genes were ranked according to MCC scores, and the ten highest-ranked genes were retained as hub genes. The resulting MCC-derived hub gene subnetwork was visualized in Cytoscape.

The methodological design of the PPI and Cytoscape-based network analyses was implemented with consideration of previously published protocols that applied comparable STRING/Cytoscape-based network pharmacology approaches [20,21,22,23,24].

### 2.11. Disease Enrichment Analysis

Disease enrichment analysis was performed using the Enrichr platform (https://maayanlab.cloud/Enrichr/; accessed on 8 June 2026) [25]. The 28 ECS–immune-associated genes were analyzed using disease-associated libraries within the Diseases/Drugs category, including Jensen DISEASES Curated 2025, DisGeNET, and OMIM Disease. Enrichment results were ranked according to adjusted *p* values, and the most informative disease-associated terms were selected for downstream analysis and visualization.

### 2.12. Drug Repurposing Analysis

Drug repurposing analysis was performed using the Drug–Gene Interaction Database (DGIdb; https://www.dgidb.org/; accessed 9 June 2026) [26]. ECS-related and immune-associated candidate genes identified through the integrative transcriptomic analyses were queried against DGIdb to identify known drug–gene interactions. Drug interaction records, regulatory approval status, therapeutic indications, and interaction scores were retrieved from the database. For downstream interpretation, only interactions involving approved therapeutic agents were retained. Representative drug–gene interactions involving key ECS-related and immune-associated targets were selected for visualization and reporting in the main manuscript. Interaction scores were reported as provided by DGIdb and were used as a relative measure of evidence supporting each drug–gene interaction. No additional filtering based on statistical significance was applied.

### 2.13. Transcription Factor and miRNA Regulatory Network Analysis

Transcription factor (TF) and miRNA regulatory annotation analyses were performed to characterize potential upstream regulatory elements associated with the 28-gene SCI–ECS immune panel. The input gene set consisted of the three ECS-related biomarker genes identified in the human cohort together with the immune marker genes used for immune-remodeling analysis. All analyses were conducted using official human gene symbols, and the results were interpreted as database-derived regulatory annotations rather than as evidence of experimentally validated regulatory activity in SCI tissue. TF enrichment analysis was performed using Enrichr (https://maayanlab.cloud/Enrichr/; accessed on 10 June 2026) [27] with the ChEA 2022 transcription factor target library [28]. The 28-gene SCI–ECS immune panel was submitted as the query gene list, and enriched TF terms were ranked according to the nominal p values, adjusted p values, odds ratios, and combined scores provided by Enrichr. Target genes contributing to each TF annotation were retained from the enrichment output. Because ChEA 2022 annotations are derived from previously reported TF–target associations, these results were used to provide regulatory context for the SCI–ECS immune panel and were not interpreted as direct measurements of TF activity in the analyzed SCI datasets. The top-ranked TF annotations were summarized in the main manuscript, whereas detailed TF–target gene information was retained for supplementary reporting.

miRNA regulatory network analysis was performed using miRNet 2.0 (https://www.mirnet.ca/; accessed on 10 June 2026), a web-based platform for miRNA-centric network visual analytics [29]. The same 28-gene SCI–ECS immune panel was submitted using *Homo sapiens* as the organism and official gene symbols as the input identifier type. Gene-to-miRNA interaction results were retrieved from the miRNet interaction table. The downloaded gene2miRNA output included miRNA identifiers, accession numbers, target genes, evidence methods, the literature PMID annotations, and tissue information when available. Unmapped genes were recorded as reported by the platform and were not manually replaced or imputed. For downstream summarization, miRNAs were ranked according to the number of unique target genes within the SCI–ECS immune panel. A main miRNA regulator table was generated from the top-ranked miRNAs and their corresponding targeted panel genes. For network visualization, the top 10 miRNAs ranked by target-gene count were connected to their target genes within the panel to generate a miRNA–target regulatory network. Nodes represented miRNAs or target genes, whereas edges represented reported miRNA–target interactions retrieved through miRNet. This network was interpreted as a regulatory annotation map and not as proof of functional miRNA-mediated regulation in SCI tissue.

### 2.14. Software and Statistical Analysis

All statistical analyses were performed in R version 4.6.0 (R Foundation for Statistical Computing, Vienna, Austria) using RStudio version 2026.04.0+526 (Posit Software, PBC, Boston, MA, USA). Differential expression analyses were conducted using the limma package (version 3.68.4) [13]. Mouse RNA-sequencing count data were analyzed using the edgeR (version 4.10.1) and voom–limma workflow (limma version 3.68.4) [13,14,15]. Data manipulation and visualization were performed using the tidyverse (version 2.0.0), ggplot2 (version 4.0.3), pheatmap (version 1.0.13), and igraph (version 2.3.2) packages. ROC analyses (version 1.19.0.1) were conducted using the pROC package [17]. Machine-learning analyses were performed using the glmnet (version 5.0) package implementing LASSO penalized logistic regression.

Protein–protein interaction (PPI), hub-gene, disease-enrichment, drug-repurposing, and regulatory annotation analyses were performed using the database and software platforms described in the corresponding Methods sections, including STRING, Cytoscape/cytoHubba, Enrichr, DGIdb, ChEA 2022, and miRNet 2.0. Results obtained from these resources were interpreted according to the statistical measures, enrichment metrics, topological scores, or interaction scores provided by each platform. Adjusted *p* values or false-discovery-rate values were used where provided by the corresponding platform. DGIdb drug–gene interaction scores were reported as database-derived evidence scores and were not treated as statistical significance values. TF and miRNA regulatory results were interpreted as database-derived regulatory annotations rather than experimentally validated regulatory activity in SCI tissue.

Continuous variables are presented as mean values unless otherwise stated. Statistical testing was performed as described in each analysis-specific Methods section. Multiple-testing correction was applied using the Benjamini–Hochberg false-discovery-rate procedure where applicable [16]. For analyses based on enrichment platforms or interaction databases, significance thresholds and ranking metrics were interpreted according to the outputs provided by the corresponding resource. All analyses were conducted using publicly available de-identified datasets, and therefore no additional institutional ethics approval or informed consent was required for the present study.

## 3. Results

### 3.1. Transcriptomic Remodeling in the Rat SCI Discovery Cohort

Genome-wide differential expression analysis of the rat discovery cohort (GSE45006) demonstrated marked temporal transcriptomic remodeling after SCI. The highest DEG burden was observed at Day 1, with 4148 DEGs, including 2430 upregulated and 1718 downregulated genes. DEG numbers decreased at Day 3 to 2663 genes, increased modestly at Week 1 to 2817 genes, and then remained at comparable lower levels at Week 2 and Week 8, with 2213 and 2214 DEGs, respectively (Supplementary Figure S1; Table 1).

### 3.2. ECS Remodeling in the Rat SCI Discovery Cohort

**Figure 2:**
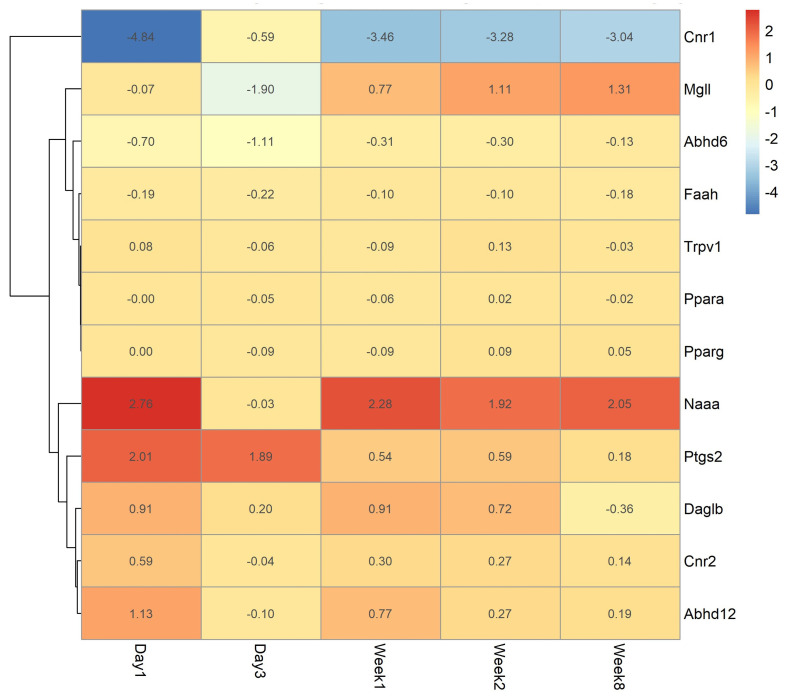
Heatmap of ECS-related gene expression changes in the rat SCI discovery cohort (GSE45006). Heatmap showing log fold-change (logFC) values of ECS-associated genes across Day 1, Day 3, Week 1, Week 2, and Week 8 after SCI relative to sham controls. Red indicates upregulation and blue indicates downregulation. *Cnr1* demonstrated persistent downregulation throughout the post-injury period, whereas *Naaa* showed sustained upregulation. *Ptgs2* exhibited early upregulation predominantly during the acute phase, while *Mgll* showed dynamic temporal remodeling characterized by early downregulation followed by positive logFC values at later time points. Hierarchical clustering was performed using Euclidean distance and complete linkage.

Focused analysis of ECS-related genes revealed a distinct temporal remodeling pattern in the rat SCI cohort. *Cnr1* showed prominent downregulation, with logFC values of −4.84 at Day 1, −3.46 at Week 1, −3.28 at Week 2, and −3.04 at Week 8; Day 3 showed a smaller change and was classified as non-significant. *Naaa* showed a sustained upregulation pattern at Day 1 (+2.76), Week 1 (+2.28), Week 2 (+1.92), and Week 8 (+2.05), with non-significance at Day 3. *Ptgs2* was upregulated during the early phase, with logFC values of +2.01 at Day 1 and +1.89 at Day 3, and was non-significant thereafter. *Mgll* showed time-dependent remodeling, with downregulation at Day 3 (−1.90) followed by later positive logFC values at Week 1 (+0.77), Week 2 (+1.11), and Week 8 (+1.31) (Figure 2; Table 2).

### 3.3. Validation of ECS Remodeling in the Mouse SCI Cohort

**Figure 3:**
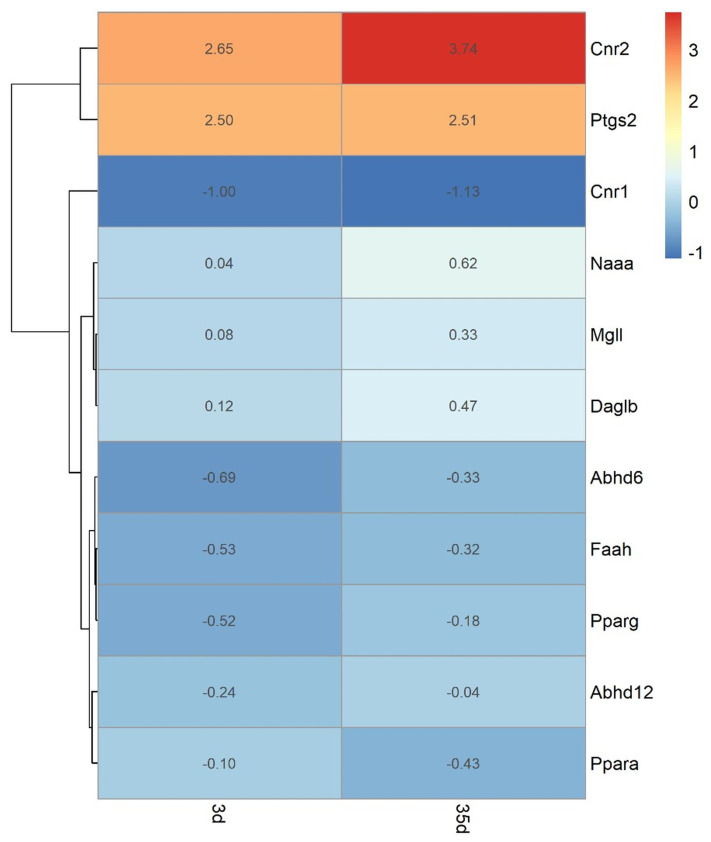
Validation of endocannabinoid-system gene remodeling in the mouse SCI cohort (GSE171441). Heatmap showing log fold-change (logFC) values of core endocannabinoid-system (ECS)-related genes at 3 days and 35 days after SCI relative to time-matched sham controls. Red indicates positive logFC values and blue indicates negative logFC values. *Cnr2* showed marked upregulation at both 3 days and 35 days, whereas *Cnr1* showed consistent downregulation. *Ptgs2* showed positive logFC values at both time points, and *Naaa* and *Daglb* displayed stronger positive changes at 35 days than at 3 days.

ECS-focused analysis in the mouse validation cohort (GSE171441) identified five significant ECS genes at 3 days after SCI: *Abhd6* (logFC −0.693, adjusted *p* = 0.002690), *Faah* (logFC −0.533, adjusted *p* = 0.005840), *Cnr2* (logFC +2.646, adjusted *p* = 0.006595), *Ptgs2* (logFC +2.498, adjusted *p* = 0.021963), and *Cnr1* (logFC −1.004, adjusted *p* = 0.026148). At 35 days after SCI, five ECS genes were significant: *Cnr2* (logFC +3.741, adjusted *p* = 0.003439), *Daglb* (logFC +0.473, adjusted *p* = 0.009055), Cnr1 (logFC −1.132, adjusted *p* = 0.016947), *Naaa* (logFC +0.621, adjusted *p* = 0.021005), and *Faah* (logFC −0.323, adjusted *p* = 0.041624) (Figure 3; Table 3).

The full mouse ECS logFC matrix showed persistent Cnr1 downregulation at both 3 days and 35 days, marked *Cnr2* upregulation at both time points, and positive *Ptgs2* logFC values at 3 days (+2.498) and 35 days (+2.515). *Ptgs2* reached statistical significance at 3 days but not at 35 days. *Naaa* and *Daglb* showed stronger positive changes at 35 days than at 3 days (Figure 3; Table 4).

Genome-wide analysis in the same mouse validation cohort identified 1972 DEGs at 3 days after SCI, including 1451 upregulated and 521 downregulated genes. At 35 days, 1787 DEGs were identified, including 1508 upregulated and 279 downregulated genes (Supplementary Figure S2; Table 5).

### 3.4. Human ECS Remodeling in Peripheral Blood After SCI

**Figure 4:**
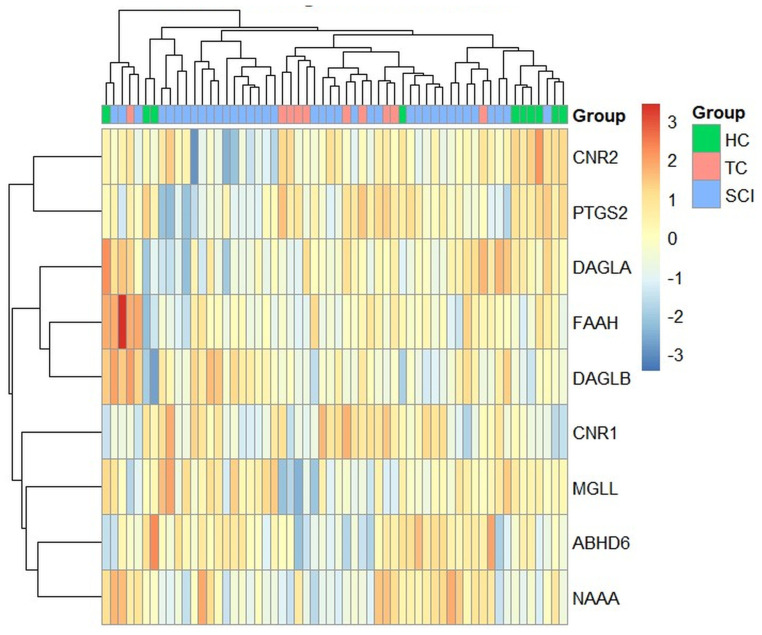
Human endocannabinoid-system gene expression profile in SCI blood (GSE151371). Heatmap showing scaled expression patterns of ECS-related genes across healthy controls (HC), non-CNS trauma controls (TC), and SCI patients. Samples are annotated by group status. The heatmap includes *CNR2*, *PTGS2*, *DAGLA*, *FAAH*, *DAGLB*, *CNR1*, *MGLL*, *ABHD6*, and *NAAA*. Differential expression analysis comparing SCI patients with healthy controls identified significant downregulation of *CNR2* and *PTGS2* and upregulation of *DAGLB*.

In the human translational cohort (GSE151371), differential expression analysis of ECS genes identified three significant alterations in SCI blood compared with healthy controls. *CNR2* was downregulated (logFC −1.471, adjusted *p* = 0.00064), *PTGS2* was downregulated (logFC −1.257, adjusted *p* = 0.00080), and DAGLB was upregulated (logFC +0.469, adjusted *p* = 0.00668). *CNR1*, *FAAH*, *MGLL*, *NAAA*, *ABHD6*, and *DAGLA* did not reach statistical significance (Figure 4; Table 6).

### 3.5. Cross-Species and Cross-Compartment ECS Remodeling After SCI

Cross-species integration identified both concordant and compartment-dependent ECS alterations across rat, mouse, and human SCI datasets. *Cnr1* showed consistent downregulation in rat and mouse spinal cord tissue but was non-significant in human blood. *Cnr2* was strongly upregulated in mouse spinal cord at both 3 days and 35 days but downregulated in human blood, while remaining non-significant in rat spinal cord. *NAAA* was upregulated in rat and mouse at 35 days but was non-significant in human blood. *PTGS2* showed acute-phase upregulation in rat spinal cord and significant upregulation in mouse spinal cord at 3 days, whereas it was downregulated in human blood. *DAGLB* was upregulated in mouse and in human blood at 35 days, representing the most concordant lipid-signaling alteration between the mouse validation and human translational cohorts (Table 7).

### 3.6. Immune Remodeling in Human SCI Blood

**Figure 5:**
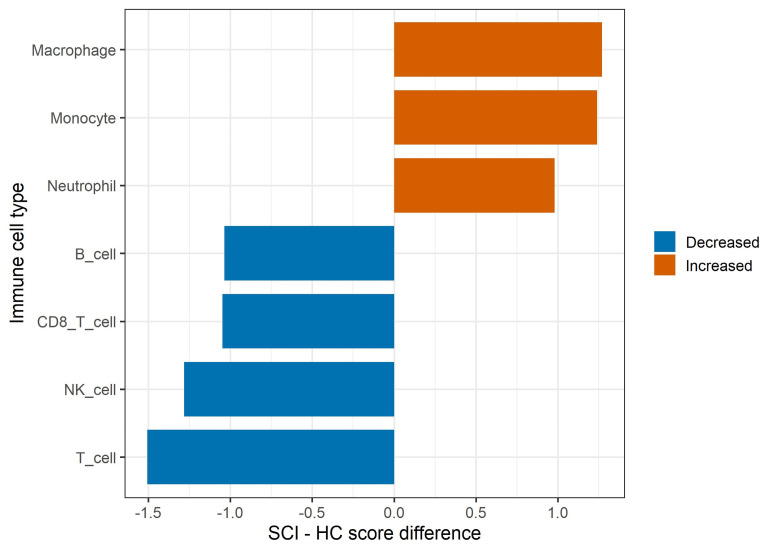
Immune remodeling in human SCI blood. Marker-based immune-cell signature scores were compared between SCI patients and healthy controls in the human translational validation cohort (GSE151371). Bars show mean SCI minus healthy-control score differences. Positive values indicate increased signatures in SCI, whereas negative values indicate decreased signatures. Monocyte, macrophage, and neutrophil signatures were increased, while T cell, NK cell, CD8 T cell, and B cell signatures were decreased.

Marker-based immune-cell scoring revealed a clear immune remodeling pattern in human SCI blood. Monocyte, macrophage, and neutrophil signatures were increased in SCI compared with healthy controls, whereas T cell, NK cell, CD8 T cell, and B cell signatures were decreased. The largest positive differences were observed for macrophages (+1.27, adjusted *p* = 2.77 × 10^−8^), monocytes (+1.24, adjusted *p* = 1.64 × 10^−17^), and neutrophils (+0.98, adjusted *p* = 3.09 × 10^−5^). The largest decreases were observed for T cells (−1.51, adjusted *p* = 2.77 × 10^−8^), NK cells (−1.28, adjusted *p* = 2.18 × 10^−3^), CD8 T cells (−1.05, adjusted *p* = 9.27 × 10^−3^), and B cells (−1.04, adjusted *p* = 3.82 × 10^−5^) (Figure 5; Table 8).

### 3.7. ECS–Immune Interaction Analysis

**Figure 6:**
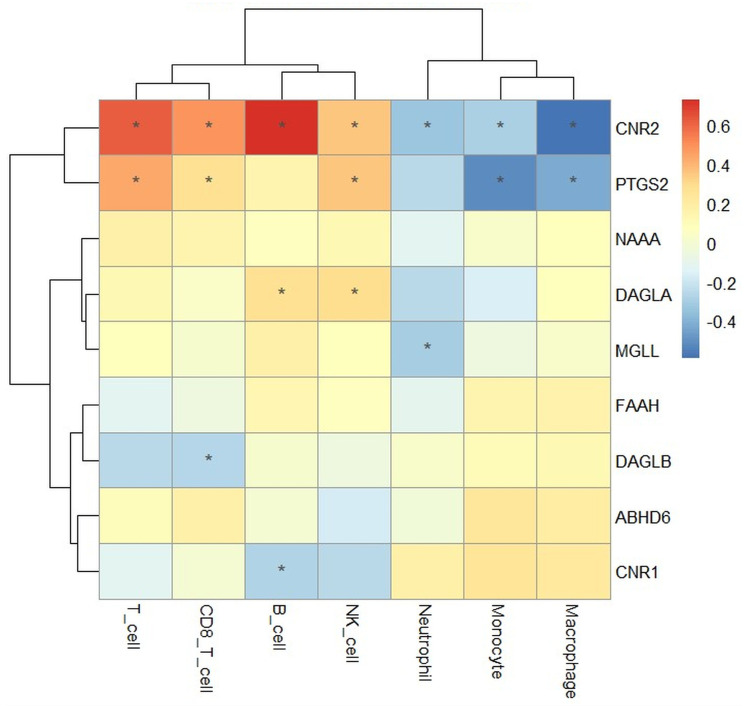
ECS–immune correlation matrix in the human SCI blood cohort. Heatmap showing Spearman correlation coefficients between ECS gene expression levels and immune-cell signature scores in the human translational cohort (GSE151371). Red indicates positive correlations and blue indicates negative correlations. Asterisks indicate statistically significant correlations. *CNR2* showed positive correlations with lymphoid immune signatures and negative correlation with macrophage signatures, whereas *PTGS2* showed negative correlations with monocyte and macrophage signatures and positive correlations with T-cell and NK-cell signatures.

Spearman correlation analysis identified significant associations between ECS gene expression and immune-cell signature scores in the human cohort. *CNR2* showed positive correlations with B-cell (ρ = 0.7318896, adjusted *p* < 1 × 10^−6^), T-cell (ρ = 0.6252730, adjusted *p* = 1.013620 × 10^−5^), and CD8 T-cell signatures (ρ = 0.4909717, adjusted *p* = 0.001407739), and a negative correlation with macrophage signatures (ρ = −0.5883601, adjusted *p* = 4.140455 × 10^−5^). *PTGS2* showed negative correlations with monocyte (ρ = −0.5118275, adjusted *p* = 0.0008106710) and macrophage signatures (ρ = −0.4176382, adjusted *p* = 0.01094716), and positive correlations with T-cell (ρ = 0.4297579, adjusted *p* = 0.008901186) and NK-cell signatures (ρ = 0.3611000, adjusted *p* = 0.04414471). These relationships are visualized in the ECS–immune correlation heatmap (Figure 6; Table 9).

### 3.8. Diagnostic Performance of ECS Biomarkers in Human SCI

**Figure 7:**
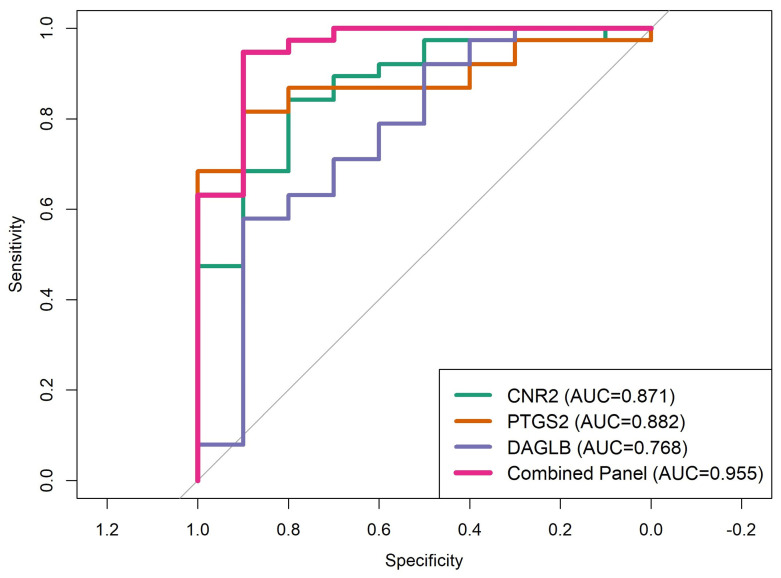
Diagnostic performance of ECS biomarkers in human SCI. Receiver operating characteristic (ROC) curves showing the discriminatory performance of individual ECS genes and the combined ECS panel for distinguishing SCI patients from healthy controls in the human translational cohort (GSE151371). *PTGS2* showed the highest individual performance (AUC = 0.882), followed by *CNR2* (AUC = 0.871) and *DAGLB* (AUC = 0.768). The combined *CNR2*–*PTGS2*–*DAGLB* panel achieved the highest performance (AUC = 0.955).

ROC analysis was performed for the three ECS genes significantly altered in human SCI blood. *PTGS2* showed the highest individual discriminatory performance, with an AUC of 0.882, followed by *CNR2* with an AUC of 0.871 and *DAGLB* with an AUC of 0.768. A combined three-gene ECS panel incorporating *CNR2*, *PTGS2*, and *DAGLB* achieved the highest performance, with an AUC of 0.955 (Figure 7).

### 3.9. LASSO-Derived ECS Machine-Learning Signature for SCI Diagnosis

To identify the most informative ECS-associated biomarkers for SCI classification, LASSO logistic regression was applied to the predefined ECS gene panel in the human translational cohort. Leave-one-out cross-validation (LOOCV) identified an optimal λ1se value of 0.0726, resulting in a parsimonious three-gene model. Among the nine ECS genes evaluated, only *CNR2*, *PTGS2*, and *DAGLB* retained non-zero coefficients and were therefore selected for the final diagnostic signature. Coefficient estimates indicated negative contributions of *CNR2* (−0.719) and *PTGS2* (−0.565), whereas *DAGLB* contributed positively (+0.791) to the model (Table 10).

**Figure 8:**
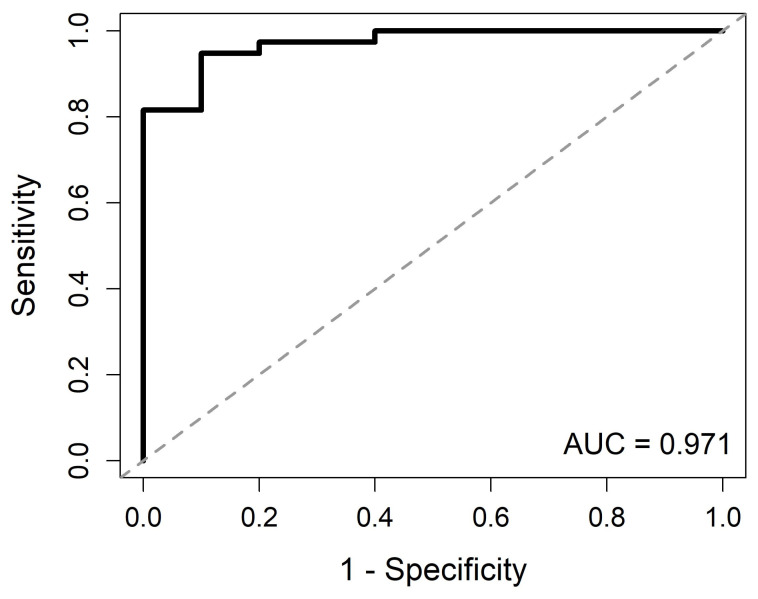
Receiver operating characteristic analysis of the LASSO-derived ECS signature for SCI diagnosis. ROC curve showing the discriminatory performance of the final LASSO-derived ECS signature for distinguishing SCI patients from healthy controls in the human translational cohort (GSE151371). The LOOCV-LASSO model retained *CNR2*, *PTGS2*, and *DAGLB* as the final three-gene signature and achieved an AUC of 0.971. The solid black line represents the ROC curve, whereas the gray dashed diagonal line indicates the line of no discrimination.

The resulting LASSO-derived ECS signature demonstrated excellent discriminatory performance for distinguishing SCI patients from healthy controls, achieving an AUC of 0.971 (Figure 8).

This performance was numerically higher than that observed for each individual ECS biomarker and the previously evaluated three-gene ECS panel. Although both multigene approaches converged on *CNR2*, *PTGS2*, and *DAGLB*, the difference in AUC values reflects the use of distinct modeling frameworks: a conventional three-gene logistic regression panel versus a regularized LASSO-derived composite signature. Cross-validation results and coefficient trajectories are presented in Supplementary Figures S3 and S4, respectively.

### 3.10. Secondary Analysis Against Non-CNS Trauma Controls

To evaluate whether the observed ECS-associated signature reflected SCI-associated biology rather than only a generalized trauma response, an additional analysis was performed comparing SCI patients with non-CNS trauma controls. *PTGS2* remained significantly dysregulated in SCI (logFC = −1.22, adjusted *p* = 0.0087), whereas *CNR2* showed a borderline trend toward differential expression (logFC = −0.92, adjusted *p* = 0.0648). *DAGLB* was not significantly altered between groups (adjusted *p* = 0.458).

**Figure 9:**
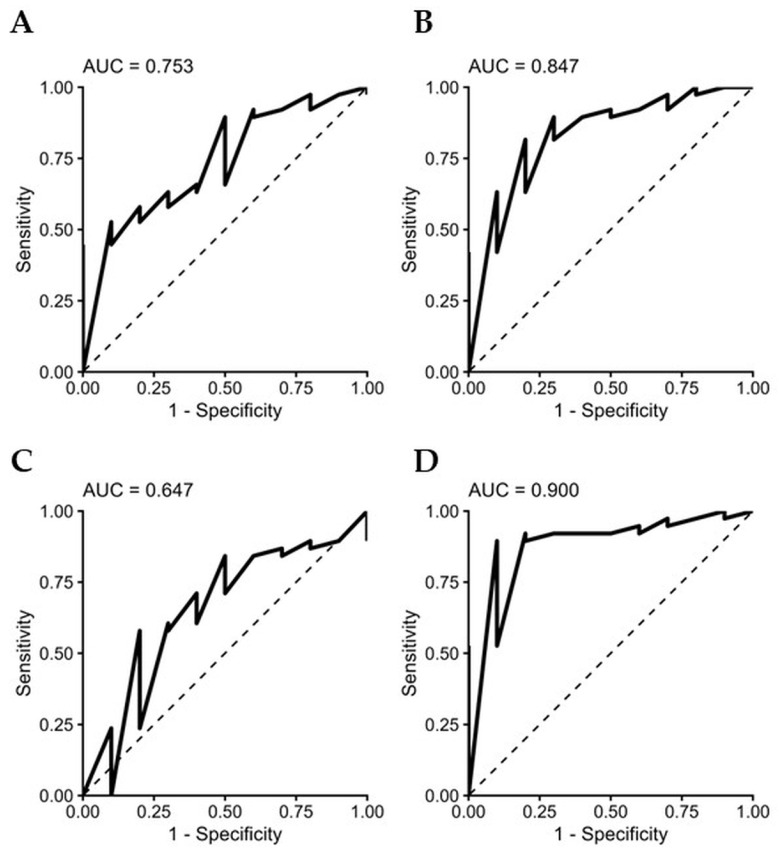
ROC performance of ECS-associated biomarkers in SCI versus non-CNS trauma controls. ROC analyses were performed for *CNR2* (**A**), *PTGS2* (**B**), *DAGLB* (**C**), and the combined *CNR2*–*PTGS2*–*DAGLB* logistic regression model (**D**). The combined ECS signature retained strong discriminatory performance between SCI patients and non-CNS trauma controls, with an AUC of 0.900. The solid black lines represent the ROC curves, whereas the dashed diagonal lines indicate the line of no discrimination.

Despite attenuation of individual-gene significance, ROC analyses demonstrated retained discriminatory performance. *PTGS2* achieved the highest classification accuracy (AUC = 0.847), followed by *CNR2* (AUC = 0.753) and *DAGLB* (AUC = 0.647). Importantly, the combined three-gene ECS signature retained strong discrimination between SCI and non-CNS trauma controls, achieving an AUC of 0.900. These findings suggest that the ECS-associated blood signature may not be explained solely by generalized trauma-associated transcriptional responses (Figure 9, Supplementary Table S6).

Consistent with ROC findings, LASSO feature selection retained *PTGS2* and *CNR2* at λ1se, whereas *DAGLB* was excluded from the final model. ROC analysis of the resulting LASSO-derived two-gene signature yielded an AUC of 0.868, supporting the persistence of ECS-associated discriminatory information beyond generalized trauma-related transcriptional responses (Supplementary Table S7).

### 3.11. PPI Network and Functional Enrichment Analysis

**Figure 10:**
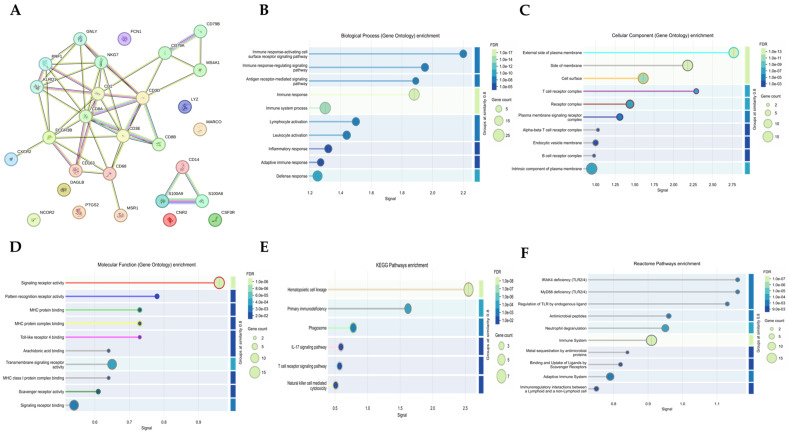
PPI network and functional enrichment analysis of the integrated ECS–immune gene set. (**A**) STRING-derived PPI network constructed from the 28-gene integrated ECS–immune input list. The network contained 28 nodes and 49 edges using a high-confidence interaction score threshold of 0.700. (**B**) GO-BP enrichment analysis. (**C**) GO-CC enrichment analysis. (**D**) GO-MF enrichment analysis. (**E**) KEGG pathway enrichment analysis. (**F**) Reactome pathway enrichment analysis. In the enrichment plots, bubble size represents the number of genes associated with each term, whereas color intensity represents FDR-adjusted significance. The x-axis represents the STRING enrichment signal.

To further characterize the biological context of the integrated ECS–immune gene set, a PPI network was constructed using the STRING database. The resulting network comprised 28 nodes and 49 edges, substantially exceeding the two interactions expected by chance. The network exhibited an average node degree of 3.5 and an average local clustering coefficient of 0.537. PPI enrichment analysis yielded a highly significant enrichment *p* value (<1.0 × 10^−16^), consistent with a non-random pattern of functional connectivity among the analyzed proteins (Figure 10A). Functional enrichment analysis indicated a predominant representation of immune-related GO-BP within the network. The most significantly enriched GO-BP terms included immune response (22 genes, FDR = 7.16 × 10^−17^), immune system process (23 genes, FDR = 2.73 × 10^−14^), immune response-activating cell surface receptor signaling pathway (9 genes, FDR = 2.49 × 10^−8^), immune response-regulating signaling pathway (10 genes, FDR = 2.49 × 10^−8^), leukocyte activation (11 genes, FDR = 2.16 × 10^−7^), antigen receptor-mediated signaling pathway (7 genes, FDR = 1.11 × 10^−6^), and inflammatory response (10 genes, FDR = 1.65 × 10^−6^) (Figure 10B; Table 11). GO-MF enrichment was characterized by signaling receptor activity (15 genes, FDR = 1.14 × 10^−6^), followed by pattern-recognition receptor activity, major histocompatibility complex (MHC) protein binding, MHC protein complex binding, Toll-like receptor 4 binding, and scavenger receptor activity (Figure 10D; Table 11). CC analysis identified significant enrichment of proteins localized to the external side of the plasma membrane (14 genes, FDR = 1.08 × 10^−13^), cell surface (15 genes, FDR = 1.17 × 10^−10^), receptor complex (9 genes, FDR = 9.42 × 10^−7^), T-cell receptor complex (4 genes, FDR = 1.82 × 10^−6^), and plasma membrane signaling receptor complex (6 genes, FDR = 4.96 × 10^−5^) (Figure 10C; Table 11).

Pathway enrichment analysis further identified multiple immune-associated signaling pathways. Within the KEGG database, hematopoietic cell lineage represented the most significantly enriched pathway (7 genes, FDR = 2.04 × 10^−8^), followed by primary immunodeficiency, phagosome, IL-17 signaling pathway, T-cell receptor signaling pathway, and natural killer cell-mediated cytotoxicity (Figure 10E; Table 11). Consistent with these findings, Reactome analysis highlighted enrichment of Immune System (17 genes, FDR = 1.88 × 10^−7^), Neutrophil Degranulation (8 genes, FDR = 2.90 × 10^−4^), Adaptive Immune System, Innate Immune System, and regulation of Toll-like receptor signaling pathways (Figure 10F; Table 11).

Observed genes indicate the number of input proteins associated with each enriched term. Strength and FDR values were obtained directly from STRING functional enrichment outputs.

Collectively, the functional enrichment landscape was characterized by immune-response pathways, leukocyte activation, antigen-receptor signaling, innate and adaptive immune processes, phagocytic pathways, and inflammatory signaling modules across the integrated ECS–immune gene set.

### 3.12. Hub Gene Identification and Network Topology Results

**Figure 11:**
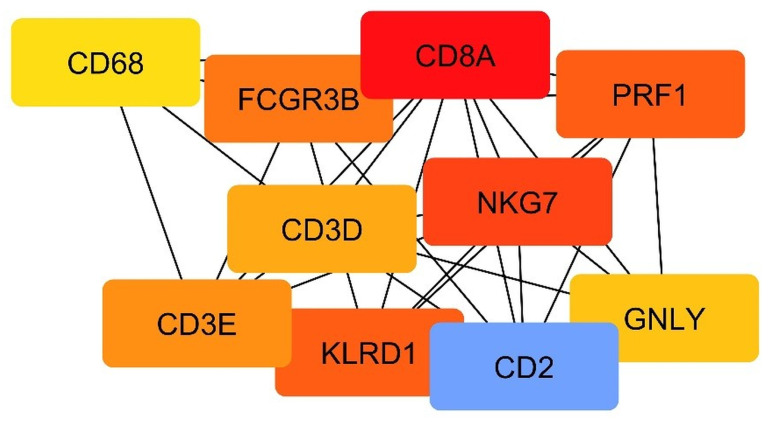
MCC-ranked hub-gene subnetwork derived from the STRING PPI network. The top 10 hub genes were selected using the MCC algorithm implemented in cytoHubba. Node colors represent MCC scores, with lower values shown in blue, intermediate values in yellow/orange, and higher values in red. Edges represent PPIs retained from the STRING-derived network.

Hub gene analysis of the integrated ECS–immune PPI network was performed using the MCC algorithm implemented in cytoHubba. The ten highest-ranked hub genes were *CD8A*, *CD2*, *NKG7*, *PRF1*, *KLRD1*, *FCGR3B*, *CD3D*, *CD68*, *CD163*, and *CD79A* (Table 12). Network topology analysis further characterized the connectivity properties of individual nodes through degree, betweenness centrality, and closeness centrality metrics (Table 13). The MCC-derived hub gene subnetwork is shown in Figure 11.

### 3.13. Disease Enrichment Results

**Figure 12:**
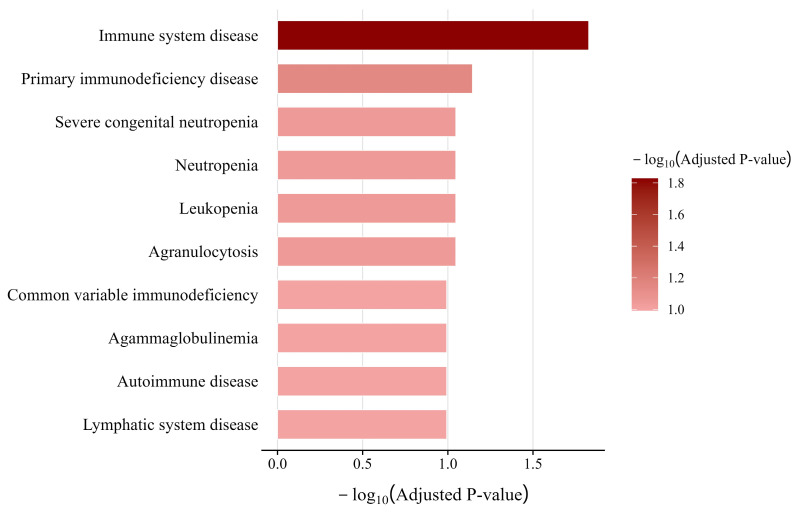
Disease enrichment analysis of the SCI–ECS immune panel using the Jensen DISEASES Curated 2025 library. The bar plot shows the top enriched disease terms identified from the predefined 28-gene SCI–ECS immune panel using Enrichr. Terms were ranked according to adjusted *p* values. Bar length and color intensity represent enrichment significance expressed as −log10(adjusted *p* value), with darker red indicating stronger enrichment. The highest-ranked annotations were predominantly immune-related, including immune system disease, primary immunodeficiency disease, severe congenital neutropenia, neutropenia, and leukopenia, supporting the immune-centered biological context of the SCI–ECS gene panel.

Disease enrichment analysis of the 28 ECS–immune-associated genes revealed a predominant association with immune-related disorders. Using the Jensen DISEASES Curated 2025 database, the most significantly enriched disease categories included immune system disease, primary immunodeficiency disease, neutropenia, leukopenia, and agranulocytosis, highlighting a strong enrichment of immune and hematological disease annotations within the analyzed gene set (Figure 12, Table 14).

To assess the robustness of disease-associated signatures across independent annotation resources, additional disease enrichment analyses were performed using the DisGeNET and OMIM Disease libraries. Although these databases identified a broader spectrum of disease terms, including autoimmune, inflammatory, hematological, immunodeficiency, and lymphoproliferative disorders, the overall enrichment pattern consistently highlighted the strong immune-related component of the ECS–immune gene signature. Notably, many enriched terms shared overlapping immune and inflammatory mechanisms, reflecting common biological processes represented within the gene set rather than direct disease specificity. Complete enrichment results obtained from the Jensen DISEASES Curated 2025, DisGeNET, and OMIM Disease databases are provided in the Supplementary Materials (Figures S5–S8 and Tables S1, S2A,B and S3).

### 3.14. Drug Repurposing Results

DGIdb analysis identified 308 drug–gene interactions across the candidate ECS-related and immune-associated targets, including 147 interactions involving approved therapeutic agents. *PTGS2* exhibited the highest number of approved drug interactions (*n* = 82), followed by *CD3E* (*n* = 17), *CD3D* (*n* = 16), *CSF3R* (*n* = 14), and *CXCR2* (*n* = 7). Representative approved drug–gene interactions are summarized in Table 15, whereas the complete list of approved interactions is provided in Supplementary Table S4.

**Figure 13:**
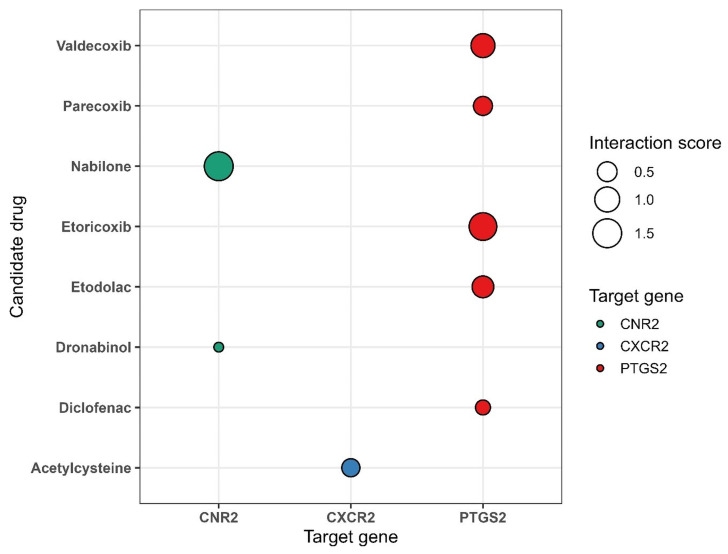
DGIdb-derived drug–gene interaction landscape of ECS-related and immune-associated pharmacologic target annotations in SCI. Bubble plot illustrating representative approved drug–gene interactions identified through DGIdb analysis. Bubble size corresponds to the DGIdb interaction score, whereas colors indicate the target gene. Among ECS-related targets, CNR2 was linked to the cannabinoid receptor agonists nabilone and dronabinol, whereas *PTGS2* was associated with several approved anti-inflammatory agents, including etoricoxib, valdecoxib, etodolac, parecoxib, and diclofenac. The immune-associated target *CXCR2* was linked to acetylcysteine. These findings provide a database-derived pharmacologic annotation context for the SCI–ECS immune signature and should be interpreted as hypothesis-generating drug–gene interaction evidence rather than evidence of therapeutic efficacy in SCI.

Among ECS-related targets, *CNR2* was linked to the cannabinoid receptor agonists nabilone and dronabinol. *PTGS2* was associated with multiple approved anti-inflammatory agents, including etoricoxib, valdecoxib, parecoxib, etodolac, and diclofenac. In addition, *CXCR2* was linked to acetylcysteine. The interaction landscape of the prioritized approved drug–gene interactions is presented in Figure 13.

### 3.15. Transcription Factor and miRNA Regulatory Annotation of the SCI–ECS Immune Panel

**Figure 14:**
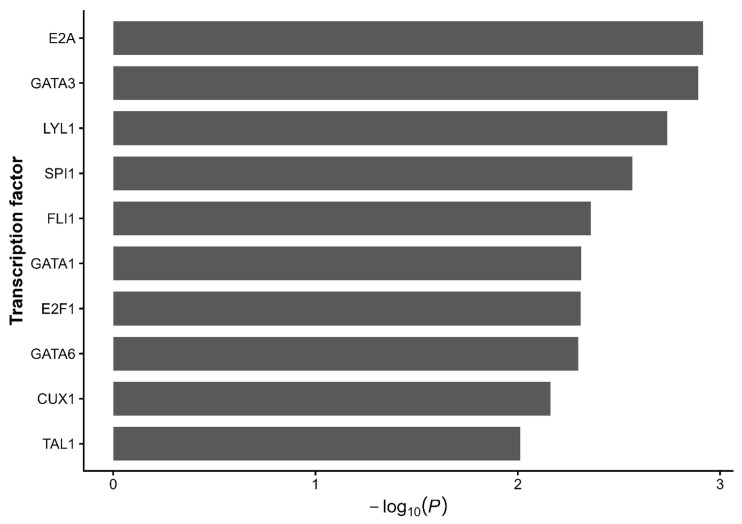
Top transcription factor annotations identified by ChEA 2022 analysis. The 28-gene SCI–ECS immune panel was submitted to the ChEA 2022 database via Enrichr to identify putative upstream transcriptional regulators. Bars represent enrichment significance expressed as −log10(nominal *p* value). *E2A*, *GATA3*, *LYL1*, *SPI1*, and *FLI1* were among the highest-ranked transcription factor annotations. Although several TFs showed nominal enrichment, none remained significant after multiple-testing correction (adjusted *p* value > 0.05).

To further contextualize the regulatory architecture of the SCI–ECS immune panel, transcription factor and miRNA regulatory annotation analyses were performed using the predefined 28-gene panel. ChEA 2022 analysis identified several transcription factor annotations with nominal enrichment, including *E2A*, *GATA3*, *LYL1*, *SPI1*, and *FLI1* among the highest-ranked terms (Figure 14; Table 16).

*E2A* showed the lowest nominal *p* value among the ranked annotations, with an overlap of 8/1603 target genes, followed by *GATA3* and *LYL1*. However, none of the TF annotations remained significant after Benjamini–Hochberg correction, as all adjusted *p* values were > 0.05. Accordingly, these findings were interpreted as exploratory regulatory annotations that may provide biological context for the SCI–ECS immune panel, but not as evidence of statistically robust TF enrichment or direct TF activity in SCI tissue. Detailed TF–target gene annotations are provided in Supplementary Table S5.

miRNA regulatory network analysis using miRNet identified multiple miRNAs connected to genes within the SCI–ECS immune panel. miRNAs were prioritized according to the number of unique panel genes targeted by each miRNA. hsa-miR-27a-3p showed the highest connectivity, targeting 11 genes within the panel, including CD14, CD163, *CD2*, *CD3E*, *CXCR2*, *DAGLB*, *MARCO*, *MS4A1*, *NCOR2*, *PTGS2*, and *S100A9* (Table 17).

miRNAs were ranked according to the number of unique target genes within the predefined SCI–ECS immune panel using miRNet-derived miRNA–target interactions. Targeted genes (n) indicates the number of panel genes associated with each miRNA.

**Figure 15:**
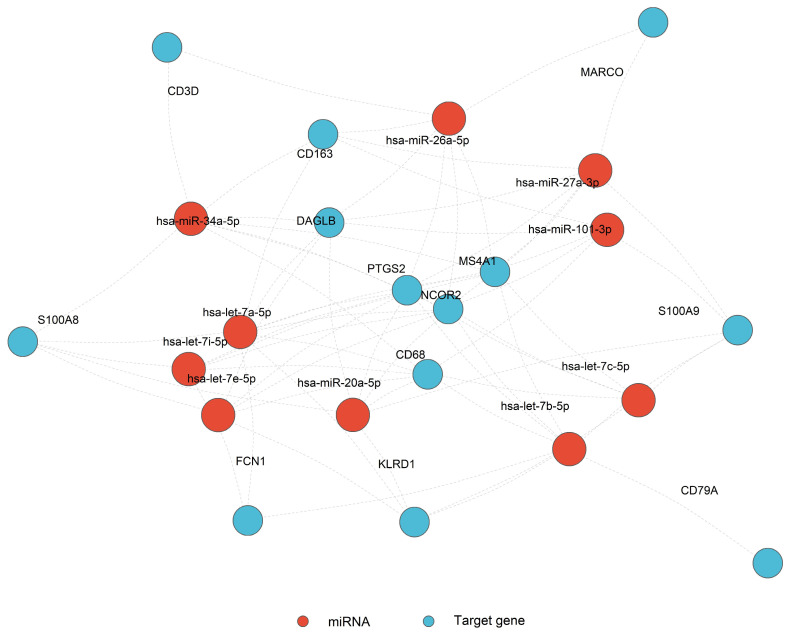
miRNA–target gene regulatory network of the SCI–ECS immune panel. The network was generated from miRNet-derived miRNA–target interactions using the predefined 28-gene SCI–ECS immune panel as input. Red nodes represent the top 10 miRNAs ranked by the number of targeted panel genes, whereas blue nodes represent target genes within the panel. Gray edges indicate reported miRNA–target interactions retrieved from miRNet interaction data. The network is presented as a regulatory annotation map and does not imply experimentally validated regulation in SCI tissue.

Four additional miRNAs—hsa-let-7a-5p, hsa-let-7b-5p, hsa-miR-20a-5p, and hsa-miR-34a-5p—each targeted 10 panel genes. Network visualization of the top 10 miRNAs demonstrated a connected miRNA–target gene regulatory map linking both ECS-related genes and immune marker genes (Figure 15).

These findings indicate that the SCI–ECS immune panel is embedded within a broader database-derived post-transcriptional regulatory network; however, they should be interpreted as regulatory annotations rather than experimentally validated miRNA-mediated regulation in SCI tissue.

## 4. Discussion

Traumatic SCI induces a sustained and multi-compartmental molecular reorganization that extends well beyond the injury epicenter and persists across acute, subacute, and chronic phases. The present study examined ECS-associated transcriptional remodeling through a cross-species discovery–validation–translation design, integrating a rat discovery cohort (GSE45006), an independent mouse validation cohort (GSE171441), and a human peripheral blood translational cohort (GSE151371).

Across these datasets, ECS-related and immune-associated transcriptional patterns showed coordinated but compartment-dependent perturbation after SCI, with *CNR2*, *PTGS2*, and *DAGLB* emerging as the most statistically prominent ECS-related genes in the human blood context. Marker-based immune scoring in the human cohort demonstrated pronounced innate immune enrichment alongside depletion of adaptive immune cell signatures, and ECS–immune correlation analysis identified significant, directionally coherent associations between ECS gene expression and immune-cell population scores.

ROC and LASSO analyses prioritized the same three-gene combination as a candidate biomarker signature with promising discriminatory performance in this cohort, a finding that should be interpreted as a translational prioritization signal rather than an indication of clinical diagnostic readiness. STRING PPI and Cytoscape network analysis placed the 28-gene SCI–ECS immune panel within a biologically coherent immune interaction landscape; DGIdb querying identified candidate drug–gene relationships for ECS-related targets; and TF and miRNA annotation analyses provided exploratory regulatory context.

Collectively, these findings suggest that ECS transcriptional remodeling and peripheral immune reorganization after SCI are linked at the transcriptomic level and may share regulatory architecture that warrants further functional investigation.

The ECS gene remodeling observed across the rat and mouse cohorts reflects a pattern broadly consistent with the known biology of CNS tissue under inflammatory stress. *CNR2* upregulation was observed in injured spinal cord tissue in a time- and dataset-dependent manner, a directional change consistent with prior reports describing induction of CB2 receptor expression in activated immune and glial populations following CNS injury and its association with neuroinflammatory signaling [30]. *PTGS2* showed early upregulation in the injured cord in both species, concordant with the recognized role of prostanoid biosynthesis in the acute post-injury inflammatory response. *DAGLB*, which encodes an enzyme implicated in 2-arachidonoylglycerol biosynthesis, showed comparatively concordant upregulation at the later time point in mice and in human blood, making it a biologically coherent lipid-signaling candidate across the mouse validation and human translational cohorts. In the human peripheral blood dataset, however, *CNR2* and *PTGS2* were downregulated relative to healthy controls, while *DAGLB* was upregulated—a pattern that diverges directionally from the spinal cord tissue findings for *CNR2* and *PTGS2*. This compartment-dependent divergence may reflect the distinct cellular compositions and signaling environments of injured CNS tissue versus peripheral leukocytes, and it highlights a recurring interpretive challenge in cross-species, cross-compartment transcriptomic integration: coordinated ECS perturbation at the system level does not imply directional uniformity across tissues or species. These observations are consistent with, though do not establish, a model in which ECS lipid signaling undergoes remodeling in parallel with the inflammatory state, with dynamics that differ by compartment and injury phase.

The immune remodeling profile identified in human SCI blood—characterized by elevation of monocyte, macrophage, and neutrophil scores alongside reductions in T cell, NK cell, CD8 T cell, and B cell signatures—is broadly consistent with the emerging body of evidence describing peripheral innate immune predominance and adaptive immune contraction after SCI. Recent clinical studies employing high-dimensional single-cell peripheral blood immunophenotyping have elaborated the complexity of this reorganization, documenting expansions of exhausted, activation-marker-bearing, and suppressive T and NK cell subsets as well as shifts in monocyte subset composition that vary with injury chronicity, severity, and the presence of neuropathic pain [31,32].

The marker-based immune scoring approach employed in the present study captures population-level directional shifts within a bulk RNA-sequencing framework and cannot resolve the subtype-specific biology characterized in those high-resolution analyses; the findings are therefore complementary rather than equivalent. Nonetheless, the magnitude and statistical significance of the innate–adaptive divergence observed here are concordant with independent immune profiling evidence, lending biological plausibility to the ECS–immune correlation results. The significant positive correlations between *CNR2* expression and lymphoid immune signatures, and the negative correlations between *CNR2* and macrophage signatures, suggest that ECS gene expression may covary with the adaptive–innate immune balance in SCI blood. Whether this reflects a direct regulatory relationship or shared upstream determinants cannot be resolved from transcriptional correlation data alone and would require cell-sorted or single-cell approaches to examine.

The discriminatory performance of the combined *CNR2*–*PTGS2*–*DAGLB* signature in ROC and LASSO analyses in the human cohort merits careful interpretive framing. The fact that LASSO-based regularization converged independently on the same three-gene set that showed individual discriminatory performance in ROC analysis is internally consistent and supports the biological relevance of these genes within the ECS panel. The analyses were performed in a single cohort of modest size using leave-one-out cross-validation, a procedure appropriate to the sample size but insufficient to establish generalizable diagnostic performance; these results are therefore best understood as biomarker prioritization evidence rather than as clinical diagnostic validation. The bulk RNA-sequencing platform integrates expression signals from heterogeneous leukocyte populations, and the apparent discriminatory utility of ECS genes likely reflects, in part, their covariation with the immune compositional shifts characterized elsewhere in this analysis; disentangling intrinsic ECS signals from immune composition-driven covariation would require cell-type-resolved approaches.

An important additional consideration when interpreting blood-based transcriptomic biomarkers in SCI is the potential contribution of generalized trauma-associated inflammatory responses. Because the GSE151371 cohort included both SCI patients and non-CNS trauma controls, we performed an additional sensitivity analysis to evaluate whether the ECS-associated signal extended beyond a generic trauma response. Although the differential-expression significance of individual genes was attenuated, *PTGS2* remained significantly dysregulated, while the combined three-gene ECS signature retained strong discriminatory performance against trauma controls (AUC = 0.900). These findings suggest that the observed ECS-associated blood signature is not fully explained by generalized trauma-related transcriptional remodeling. At the same time, the partial attenuation observed for *CNR2* and the loss of significance for *DAGLB* indicate that some components of the peripheral ECS response may overlap with broader injury-associated inflammatory programs. Therefore, the proposed ECS signature should be interpreted as reflecting both SCI-related and systemic injury-related biological processes rather than a purely SCI-specific molecular fingerprint.

The STRING PPI network analysis demonstrated that the 28-gene SCI–ECS immune panel forms a statistically non-random interaction network with functional enrichment dominated by immune response pathways, leukocyte activation, antigen-receptor signaling, innate and adaptive immune processes, and phagocytic biology. This enrichment landscape provides a systems-level biological context for the panel but primarily reflects its immune composition and should not be interpreted as evidence of SCI-specific pathway activation.

Hub genes identified through Cytoscape MCC analysis were predominantly cytotoxic and myeloid lineage markers, consistent with the immune biology captured by the input gene set. Disease enrichment analysis consistently highlighted immune, autoimmune, immunodeficiency, and hematological annotations; the absence of SCI-specific terms is expected given the panel’s immune-centric composition and confirms the inflammatory–immune biological context of the signature rather than implying disease specificity. DGIdb analysis identified candidate drug–gene relationships for ECS-related targets within the panel—including cannabinoid receptor agonists linked to *CNR2* and COX-2-directed anti-inflammatory agents linked to *PTGS2*—that provide a database-derived pharmacological context for these targets.

These interactions represent hypothesis-generating observations from a drug–gene interaction database and should not be interpreted as evidence of clinical therapeutic efficacy in SCI.

TF enrichment analysis using ChEA 2022 identified several nominally enriched candidate regulators of the 28-gene panel, including *E2A*, *GATA3*, *LYL1*, *SPI1*, and *FLI1*—transcription factors with established roles in hematopoietic specification, lymphoid development, and myeloid lineage regulation. These annotations are biologically plausible in the context of an immune-centric input gene set; however, none survived Benjamini–Hochberg multiple-testing correction, and all adjusted *p* values exceeded 0.05. ChEA 2022 enrichment results derive from aggregated prior TF–target association datasets rather than from measurements made in SCI tissue, and the nominal signals observed here should therefore be interpreted as exploratory regulatory annotations that may inform hypothesis generation rather than as evidence of active transcriptional regulation in the present SCI datasets. miRNA regulatory annotation via miRNet identified multiple broadly connected miRNAs—including hsa-miR-27a-3p, hsa-let-7 family members, and hsa-miR-34a-5p—as candidate regulators of multiple panel genes based on curated database-derived miRNA–target interactions. Their broad connectivity within the panel is consistent with the general architecture of database-derived post-transcriptional regulatory networks involving immune-relevant gene sets. The miRNet results are database-derived annotations and do not constitute experimental evidence of miRNA-mediated regulation in SCI tissue or SCI blood; the distinction between regulatory annotation and functional validation is critical and should not be conflated in the interpretation of these findings.

Beyond molecular biomarker and pharmacologic-target annotation, the present findings should be interpreted within the broader clinical and systemic-management context of SCI. SCI is increasingly recognized as a multi-system disorder involving bidirectional communication among the injured nervous system, systemic immunity, and the gastrointestinal tract. Neurologic injury may be associated with gut dysbiosis, impaired intestinal barrier integrity, altered gastrointestinal motility, and downstream immune-inflammatory consequences, supporting the relevance of gut-health optimization as part of comprehensive SCI care [33]. Although the present transcriptomic analysis did not directly evaluate gut microbiota, nutrition, or bowel-function parameters, the peripheral immune remodeling observed here is consistent with the broader concept that systemic immune and barrier-related processes may contribute to post-injury biology. Future studies integrating blood transcriptomics with microbiome profiling, metabolomics, nutritional status, and bowel-function data may help clarify how gut–immune interactions influence SCI outcomes.

Respiratory management is another clinically important determinant of outcome in acute traumatic SCI, particularly in patients with cervical or high thoracic injuries and anticipated prolonged ventilatory support. Recent systematic-review and meta-analytic evidence suggests that early tracheostomy, commonly defined as tracheostomy performed within 7 days of injury, surgery, or endotracheal intubation, may reduce mechanical-ventilation duration, ICU length of stay, hospital length of stay, and tracheostomy-related complications in acute traumatic SCI [34]. These considerations do not alter the transcriptomic conclusions of the present study, but they emphasize that peripheral blood molecular signatures should be interpreted within the broader clinical context of SCI, including systemic complications, ventilatory support, and tracheostomy timing.

Several limitations of the present study require explicit acknowledgment. All analyses relied on publicly available transcriptomic datasets, and the results are contingent on the quality, processing, and representativeness of these resources. The rat and mouse cohorts reflect spinal cord tissue biology, whereas the human cohort profiles peripheral blood, making direct cross-compartment comparisons inherently uncertain and requiring cautious interpretation. Bulk RNA-sequencing aggregates expression signals from heterogeneous cell populations, and marker-based immune scoring captures directional population-level shifts rather than true cellular deconvolution; inferred immune signatures may not fully reflect underlying cellular composition. Cross-species comparisons are further complicated by differences in injury model, sequencing platform, tissue compartment, and post-injury sampling intervals. An additional consideration when interpreting the cross-species findings is the substantial temporal heterogeneity among the analyzed datasets. The rat discovery cohort spans acute, subacute, and chronic stages after SCI, the mouse validation cohort includes 3- and 35-day post-injury time points, whereas the human translational cohort represents acute-phase peripheral blood samples. Because inflammatory and ECS-associated signaling pathways undergo dynamic remodeling throughout the post-injury course, direct comparisons across datasets should be interpreted cautiously. Accordingly, the cross-species analyses performed in the present study should be interpreted as identifying broad ECS-associated remodeling patterns rather than temporally synchronized molecular signatures. The human SCI cohort encompasses patients who vary in injury level, severity, age, and time from injury to blood collection, and this biological heterogeneity is not fully accounted for in the present analyses. All PPI, disease enrichment, drug–gene interaction, TF, and miRNA relationships reported here are database-derived and represent catalogued associations rather than experimentally validated interactions in SCI-relevant systems. The modest size of the human cohort limits the generalizability of the biomarker performance estimates, and cross-validation cannot substitute for external prospective validation.

Moving forward, prospective validation in larger and independently characterized human SCI cohorts is needed to assess the reproducibility of the ECS–immune signature. Experimental studies in appropriate cellular and animal models are required to evaluate functional ECS–immune interactions in the post-injury context. Cell-type-resolved transcriptomic analyses—including single-cell RNA sequencing and spatial multi-omics approaches that have recently been applied to characterize the cellular and molecular landscape of the SCI microenvironment [35]—would provide the resolution necessary to disentangle ECS and immune cell-specific contributions, and formal experimental testing of the TF and miRNA regulatory relationships annotated here is warranted before any mechanistic conclusions can be drawn.

## 5. Conclusions

This cross-species transcriptomic study identifies a coordinated but compartment-dependent ECS-associated immune remodeling signature in SCI. By integrating rat spinal cord discovery data, independent mouse validation data, and human peripheral blood transcriptomics, the analysis highlighted *CNR2*, *PTGS2*, and *DAGLB* as key ECS-related genes associated with immune transcriptional remodeling in SCI. In the human cohort, these genes showed biomarker-prioritization potential, and their expression patterns were associated with innate–adaptive immune imbalance in peripheral blood. The combined ECS signature also retained discriminatory performance against non-CNS trauma controls, suggesting that the signature is not fully explained by generalized trauma-associated transcriptional responses. Network, disease enrichment, drug–gene interaction, TF, and miRNA annotation analyses further placed the 28-gene SCI–ECS immune panel within an immune-centered systems-level context and identified candidate pharmacologic and regulatory relationships. These findings should be interpreted as exploratory and hypothesis-generating rather than as evidence of causality, clinical diagnostic readiness, or therapeutic efficacy. Prospective validation in larger human SCI cohorts, together with cell-type-resolved and functional experimental studies, is required to confirm the biological and translational significance of the proposed ECS–immune signature.

## Figures and Tables

**Table 1:** Differentially expressed genes identified across post-SCI time points in the rat SCI discovery cohort (GSE45006). Numbers of significantly upregulated, downregulated, and total differentially expressed genes (DEGs) identified at Day 1, Day 3, Week 1, Week 2, and Week 8 after SCI relative to sham controls (adjusted *p* < 0.05).

Time Point	Upregulated	Downregulated	Total DEGs
Day 1	2430	1718	4148
Day 3	1655	1008	2663
Week 1	1607	1210	2817
Week 2	1107	1106	2213
Week 8	1157	1057	2214

**Table 2:** Temporal expression changes in ECS-related genes in the rat SCI discovery cohort (GSE45006). Log fold-change (logFC) values of ECS-associated genes at Day 1, Day 3, Week 1, Week 2, and Week 8 after SCI relative to sham controls. Positive values indicate upregulation and negative values indicate downregulation.

Gene	Day1	Day3	Week1	Week2	Week8
*Cnr1*	−4.84	−0.59	−3.46	−3.28	−3.04
*Naaa*	2.76	−0.03	2.28	1.92	2.05
*Ptgs2*	2.01	1.89	0.54	0.59	0.18
*Mgll*	−0.07	−1.90	0.77	1.11	1.31

**Table 3:** Significantly altered endocannabinoid-system (ECS)-related genes in the mouse SCI validation cohort (GSE171441). Differential expression analysis identified significant ECS-associated genes at 3 days (3 d) and 35 days (35 d) after SCI relative to sham controls. Log fold-change (logFC), nominal *p* values, and Benjamini–Hochberg FDR-adjusted *p* values are shown for all significantly differentially expressed ECS genes.

Timepoint	Gene Symbol	logFC	*p* Value	Adjusted *p* Value
3 d	*Abhd6*	−0.693	0.000195	0.002690
3 d	*Faah*	−0.533	0.000644	0.005840
3 d	*Cnr2*	2.646	0.000781	0.006595
3 d	*Ptgs2*	2.498	0.004977	0.021963
3 d	*Cnr1*	−1.004	0.006452	0.026148
35 d	*Cnr2*	3.741	0.000146	0.003439
35 d	*Daglb*	0.473	0.000705	0.009055
35 d	*Cnr1*	−1.132	0.002089	0.016947
35 d	*Naaa*	0.621	0.003071	0.021005
35 d	*Faah*	−0.323	0.011101	0.041624

**Table 4:** Expression changes in core endocannabinoid-system genes in the mouse SCI validation cohort (GSE171441). Log fold-change (logFC) values are shown for ECS-related genes at 3 days and 35 days after SCI relative to time-matched sham controls. Positive values indicate upregulation and negative values indicate downregulation.

Gene Symbol	3 d logFC	35 d logFC
*Cnr1*	−1.004	−1.132
*Cnr2*	2.646	3.741
*Faah*	−0.533	−0.323
*Mgll*	0.085	0.333
*Daglb*	0.117	0.473
*Abhd6*	−0.693	−0.327
*Abhd12*	−0.243	−0.037
*Naaa*	0.041	0.621
*Ptgs2*	2.498	2.515
*Ppara*	−0.101	−0.427
*Pparg*	−0.519	−0.182

**Table 5:** Differentially expressed genes identified in the mouse SCI validation cohort (GSE171441). Numbers of significantly upregulated, downregulated, and total differentially expressed genes (DEGs) identified at 3 days and 35 days after SCI relative to time-matched sham controls.

Timepoint	Upregulated	Downregulated	Total DEGs
3 d	1451	521	1972
35 d	1508	279	1787

**Table 6:** Human ECS logFC matrix in the translational cohort (GSE151371). Differential expression results for ECS-related genes in SCI patients compared with healthy controls. Log fold-change (logFC), Benjamini–Hochberg adjusted *p* values, and regulation direction are shown. Genes classified as ns did not reach statistical significance.

Gene	logFC	adj. *p*.Val	Regulation
*CNR2*	−1.471	0.00064	Downregulated
*PTGS2*	−1.257	0.00080	Downregulated
*DAGLB*	0.469	0.00668	Upregulated
*CNR1*	0.973	0.221	Ns
*FAAH*	0.271	0.387	Ns
*MGLL*	−0.209	0.528	Ns
*NAAA*	0.095	0.758	Ns
*ABHD6*	−0.047	0.834	Ns
*DAGLA*	−0.074	0.859	Ns

**Table 7:** Cross-species endocannabinoid-system signature after SCI. Comparative summary of ECS-related gene alterations across the rat discovery cohort (GSE45006), mouse validation cohort (GSE171441), and human translational validation cohort (GSE151371). Directional changes and logFC values are shown where applicable. The table summarizes shared, species-specific, and compartment-dependent ECS alterations across spinal cord tissue and human peripheral blood. ↑ indicates upregulation, ↓ indicates downregulation, and ns indicates non-significant change.

Gene	Rat (GSE45006)	Mouse 3 d (GSE171441)	Mouse 35 d (GSE171441)	Human Blood (GSE151371)	Cross-Species Interpretation
*CNR1*	↓	↓ (−1.004)	↓ (−1.132)	ns (+0.973)	Consistent CNS downregulation in rat and mouse
*CNR2*	ns	↑ (+2.646)	↑ (+3.741)	↓ (−1.471)	Opposite regulation in CNS tissue and peripheral blood
*FAAH*	ns	↓ (−0.533)	↓ (−0.323)	ns (+0.271)	Mouse-specific suppression
*MGLL*	Remodeling	ns (+0.085)	ns (+0.333)	ns (−0.209)	Dynamic ECS lipid metabolism without shared differential expression across cohorts
*NAAA*	↑	ns (+0.041)	↑ (+0.621)	ns (+0.095)	Shared CNS-associated activation pattern
*PTGS2*	↑	↑ (+2.498)	ns (+2.515)	↓ (−1.257)	Acute CNS inflammatory activation, opposite blood response
*DAGLB*	ns	ns (+0.117)	↑ (+0.473)	↑ (+0.469)	Most consistent lipid-signaling alteration across species
*ABHD6*	ns	↓ (−0.693)	ns (−0.327)	ns (−0.047)	Early mouse-specific response

**Table 8:** Immune cell remodeling in human SCI blood (GSE151371). Marker-based immune-cell signature scores were compared between SCI patients and healthy controls. Differences are shown as mean SCI minus healthy-control score differences. Positive values indicate increased immune-cell signatures in SCI, whereas negative values indicate decreased signatures. Benjamini–Hochberg adjusted *p* values are shown.

Cell Type	SCI-HC Difference	adj. *p*
Monocyte	+1.24	1.64 × 10^−17^
Macrophage	+1.27	2.77 × 10^−8^
Neutrophil	+0.98	3.09 × 10^−5^
T cell	−1.51	2.77 × 10^−8^
NK cell	−1.28	2.18 × 10^−3^
CD8 T cell	−1.05	9.27 × 10^−3^
B cell	−1.04	3.82 × 10^−5^

**Table 9:** ECS–immune correlation matrix in the human SCI blood cohort (GSE151371). Spearman correlation analysis was performed between endocannabinoid-system (ECS) gene expression levels and immune-cell signature scores in the human translational cohort. Correlation coefficients (ρ), nominal *p* values, Benjamini–Hochberg FDR-adjusted *p* values, and direction of association are shown for all statistically significant ECS–immune relationships.

Gene	Immune Cell Type	Spearman Rho	*p* Value	Adjusted *p* Value	Direction
*CNR2*	B cell	0.7318896	<1 × 10^−6^	<1 × 10^−6^	Positive
*CNR2*	T cell	0.6252730	3.217841 × 10^−7^	1.013620 × 10^−5^	Positive
*CNR2*	Macrophage	−0.5883601	1.971645 × 10^−6^	4.140455 × 10^−5^	Negative
*PTGS2*	Monocyte	−0.5118275	5.147118 × 10^−5^	8.106710 × 10^−4^	Negative
*CNR2*	CD8 T cell	0.4909717	1.117253 × 10^−4^	1.407739 × 10^−3^	Positive
*PTGS2*	T cell	0.4297579	8.477320 × 10^−4^	8.901186 × 10^−3^	Positive
*PTGS2*	Macrophage	−0.4176382	1.216351 × 10^−3^	1.094716 × 10^−2^	Negative
*PTGS2*	NK cell	0.3611000	5.605677 × 10^−3^	4.414471 × 10^−2^	Positive

**Table 10:** Genes retained in the LASSO-derived ECS diagnostic signature. Non-zero coefficients from the λ1se LASSO logistic-regression model are shown for ECS genes retained in the final diagnostic signature. Negative coefficients indicate inverse contributions to the model score, whereas positive coefficients indicate positive contributions.

Gene	Coefficient
*CNR2*	−0.719
*PTGS2*	−0.565
*DAGLB*	+0.791

**Table 11:** Representative enriched Gene Ontology terms and pathway annotations identified from STRING functional enrichment analysis of the integrated ECS–immune gene set.

Category	Term/Pathway	Observed Genes	Strength	FDR
GO BP	Immune response	22	1.07	7.16 × 10^−17^
GO BP	Immune system process	23	0.88	2.73 × 10^−14^
GO BP	Defense response	17	0.93	1.61 × 10^−9^
GO BP	Immune response-activating cell surface receptor signaling pathway	9	1.51	2.49 × 10^−8^
GO BP	Immune response-regulating signaling pathway	10	1.35	2.49 × 10^−8^
GO BP	Leukocyte activation	11	1.13	2.16 × 10^−7^
GO BP	Antigen receptor-mediated signaling pathway	7	1.54	1.11 × 10^−6^
GO BP	Inflammatory response	10	1.12	1.65 × 10^−6^
GO BP	Adaptive immune response	8	1.20	1.67 × 10^−5^
GO MF	Signaling receptor activity	15	0.85	1.14 × 10^−6^
GO MF	Pattern recognition receptor activity	3	1.88	0.0111
GO MF	MHC protein binding	3	1.81	0.0146
GO MF	MHC protein complex binding	3	1.78	0.0147
GO MF	Toll-like receptor 4 binding	2	2.55	0.0179
GO MF	Scavenger receptor activity	3	1.64	0.0279
GO CC	External side of plasma membrane	14	1.40	1.08 × 10^−13^
GO CC	Cell surface	15	1.07	1.17 × 10^−10^
GO CC	Receptor complex	9	1.18	9.42 × 10^−7^
GO CC	T-cell receptor complex	4	2.30	1.82 × 10^−6^
GO CC	Plasma membrane signaling receptor complex	6	1.34	4.96 × 10^−5^
KEGG Pathway	Hematopoietic cell lineage	7	1.74	2.04 × 10^−8^
KEGG Pathway	Primary immunodeficiency	4	1.88	5.33 × 10^−5^
KEGG Pathway	Phagosome	4	1.30	0.0056
KEGG Pathway	IL-17 signaling pathway	3	1.37	0.0264
KEGG Pathway	T-cell receptor signaling pathway	3	1.32	0.0277
KEGG Pathway	Natural killer cell-mediated cytotoxicity	3	1.25	0.0387
Reactome Pathway	Immune System	17	0.78	1.88 × 10^−7^
Reactome Pathway	Neutrophil degranulation	8	1.07	0.00029
Reactome Pathway	Adaptive Immune System	9	0.92	0.00052
Reactome Pathway	Innate Immune System	10	0.83	0.00055
Reactome Pathway	Regulation of TLR by endogenous ligand	3	2.02	0.0015
Reactome Pathway	Immunoregulatory interactions between a lymphoid and a non-lymphoid cell	4	1.34	0.0076

**Table 12:** Top 10 hub genes identified from the PPI network using the MCC algorithm implemented in cytoHubba.

Rank	Gene	MCC
1	*CD8A*	138
2	*CD2*	86
3	*NKG7*	78
4	*PRF1*	72
5	*KLRD1*	72
6	*FCGR3B*	48
7	*CD3D*	46
8	*CD68*	19
9	*CD163*	12
10	*CD79A*	6

**Table 13:** Degree, betweenness, and closeness centrality values of connected nodes in the PPI network calculated using Cytoscape NetworkAnalyzer; only nodes with at least one network connection are shown.

Gene	Degree	Betweenness	Closeness
*CD3D*	9	54.7667	12.3333
*CD2*	9	36.5000	12.3333
*CD68*	6	33.4667	10.8333
*CD8A*	11	33.6000	13.3333
*CD79A*	4	24.1000	9.6667
*FCGR3B*	7	9.4000	11.3333
*NKG7*	7	3.8667	11.0000
*PRF1*	6	2.5333	10.5000
*KLRD1*	6	2.5333	10.5000
*CD163*	4	0.8000	9.6667
*CD8B*	3	0.0000	9.0000
*MSR1*	2	0.0000	6.8333
*S100A8*	2	0.0000	2.0000
*S100A9*	2	0.0000	2.0000

**Table 14:** Summary of the most significant disease associations identified by disease enrichment analysis across the Jensen DISEASES Curated 2025, DisGeNET, and OMIM databases. Disease terms consistently indicated enrichment of immune-related, immunodeficiency-associated, autoimmune, and hematological disorders among the 28 ECS–immune-associated genes. Adjusted *p* values correspond to database-specific multiple-testing correction procedures.

Disease Term	Database	Adjusted *p* Value
Immune system disease	Jensen DISEASES Curated 2025	0.0149
Primary immunodeficiency disease	Jensen DISEASES Curated 2025	0.0717
Immunodeficiency	OMIM Disease	3.15 × 10^−5^
Rheumatoid arthritis	DisGeNET	3.80 × 10^−12^
Autoimmune diseases	DisGeNET	1.00 × 10^−8^
Lymphoma	DisGeNET	7.68 × 10^−10^

**Table 15:** Biologically prioritized approved drug candidates identified by DGIdb analysis.

Target Gene	Candidate Drug	Interaction Score
*CNR2*	Nabilone	1.506
*CNR2*	Dronabinol	0.118
*PTGS2*	Etoricoxib	1.339
*PTGS2*	Valdecoxib	0.892
*PTGS2*	Etodolac	0.669
*PTGS2*	Parecoxib	0.446
*PTGS2*	Diclofenac	0.223
*CXCR2*	Acetylcysteine	0.387

Note: The table highlights representative approved drug–gene interactions involving the principal ECS-related and immune-associated targets identified in this study. Complete approved DGIdb interaction results are provided in Supplementary Table S4.

**Table 16:** Top transcription factor annotations identified by ChEA 2022.

Rank	TF	Overlap	*p* Value	Adjusted *p* Value	Odds Ratio	Combined Score
1	*E2A*	8/1603	0.001213	0.280826	4.608652	30.94400
2	*GATA3*	8/1617	0.001284	0.280826	4.565071	30.39300
3	*LYL1*	6/960	0.001827	0.280826	5.436821	34.27811
4	*SPI1*	10/2723	0.002719	0.289892	3.534218	20.87865
5	*FLI1*	7/1534	0.004349	0.289892	4.026413	21.89518
6	*GATA1*	5/810	0.004870	0.289892	5.176073	27.56098
7	*E2F1*	7/1567	0.004892	0.289892	3.934188	20.93085
8	*GATA6*	7/1575	0.005031	0.289892	3.912415	20.70530
9	*CUX1*	7/1669	0.006902	0.353538	3.672282	18.27305
10	*TAL1*	5/957	0.009742	0.449091	4.343259	20.11512

Abbreviations: TF, transcription factor. Results are ranked according to nominal *p* values. Adjusted *p* values were calculated using the Benjamini–Hochberg procedure. None of the identified TF annotations remained significant after multiple-testing correction.

**Table 17:** Top miRNA regulators of the SCI–ECS immune panel.

miRNA	Targeted Genes (n)	Target Genes
hsa-miR-27a-3p	11	*CD14*; *CD163*; *CD2*; *CD3E*; *CXCR2*; *DAGLB*; *MARCO*; *MS4A1*; *NCOR2*; *PTGS2*; *S100A9*
hsa-let-7a-5p	10	*CD163*; *CD68*; *CD8A*; *DAGLB*; *FCN1*; *KLRD1*; *MS4A1*; *NCOR2*; *PTGS2*; *S100A8*
hsa-let-7b-5p	10	*CD68*; *CD79A*; *CD79B*; *CD8A*; *FCN1*; *KLRD1*; *MS4A1*; *NCOR2*; *PTGS2*; *S100A9*
hsa-miR-20a-5p	10	*CD14*; *CD2*; *CD68*; *DAGLB*; *KLRD1*; *MSR1*; *NCOR2*; *PTGS2*; *S100A8*; *S100A9*
hsa-miR-34a-5p	10	*CD14*; *CD163*; *CD2*; *CD3D*; *CD68*; *DAGLB*; *MS4A1*; *NCOR2*; *PTGS2*; *S100A8*
hsa-miR-15a-5p	9	*CD14*; *CD163*; *CD3E*; *CD68*; *CD79B*; *DAGLB*; *MS4A1*; *NCOR2*; *PTGS2*
hsa-miR-16-5p	9	*CD14*; *CD163*; *CD3E*; *CD79A*; *CD79B*; *CSF3R*; *DAGLB*; *NCOR2*; *PTGS2*
hsa-miR-26a-5p	9	*CD163*; *CD3D*; *CD8B*; *CXCR2*; *DAGLB*; *MARCO*; *MS4A1*; *NCOR2*; *PTGS2*
hsa-miR-103a-3p	8	*CD14*; *CD163*; *CD3E*; *CD68*; *CSF3R*; *MS4A1*; *NCOR2*; *PTGS2*
hsa-miR-15b-5p	8	*CD14*; *CD163*; *CD3E*; *CD68*; *CD79B*; *MS4A1*; *NCOR2*; *PTGS2*
hsa-miR-335-5p	8	*CD14*; *CD79A*; *CD8A*; *CXCR2*; *DAGLB*; *NCOR2*; *NKG7*; *PTGS2*
hsa-let-7c-5p	7	*CD68*; *CD8A*; *KLRD1*; *MS4A1*; *NCOR2*; *PTGS2*; *S100A9*
hsa-let-7g-5p	7	*CD68*; *CD79B*; *CD8A*; *FCN1*; *NCOR2*; *PTGS2*; *S100A8*
hsa-let-7i-5p	7	*CD68*; *CD8A*; *FCN1*; *MS4A1*; *NCOR2*; *PTGS2*; *S100A8*
hsa-miR-18a-5p	7	*CD14*; *CD68*; *CNR2*; *MSR1*; *NCOR2*; *PTGS2*; *S100A9*

## Data Availability

The datasets analyzed in this study are publicly available from the NCBI Gene Expression Omnibus (GEO) database under accession numbers GSE45006, GSE171441, and GSE151371. No new sequencing or transcriptomic datasets were generated in the present study. Additional supporting results generated during the analyses are provided in the Supplementary Materials.

## Supplementary Materials

The following supporting information can be downloaded at https://www.mdpi.com/article/10.3390/biomedicines14071446/s1. Figure S1: Temporal transcriptomic remodeling after SCI in the rat discovery cohort (GSE45006); Figure S2: Temporal transcriptomic remodeling in the mouse SCI validation cohort (GSE171441); Figure S3: Leave-one-out cross-validation curve used for LASSO model selection; Figure S4: LASSO coefficient trajectories for ECS-related genes across the regularization path; Figure S5: DisGeNET disease enrichment bar plot of the 28 ECS–immune-associated genes; Figure S6: Gene–disease association clustergram based on DisGeNET enrichment analysis; Figure S7: OMIM Disease enrichment bar plot of the 28 ECS–immune-associated genes; Figure S8: Gene–disease association heatmap based on OMIM Disease enrichment analysis; Table S1: Complete disease enrichment results generated using the Jensen DISEASES Curated 2025 database in Enrichr for the 28 ECS–immune-associated genes; Table S2A: Complete disease enrichment results generated using the DisGeNET database in Enrichr for the 28 ECS–immune-associated genes; Table S2B: Contributing genes for DisGeNET disease enrichment terms; Table S3: Complete disease enrichment results generated using the OMIM Disease database in Enrichr for the 28 ECS–immune-associated genes; Table S4: Complete list of approved drug–gene interactions identified by DGIdb analysis; Table S5: Detailed ChEA 2022 transcription factor annotations and overlapping target genes; Table S6: Secondary SCI-versus-non-CNS-trauma sensitivity analysis of ECS-associated biomarkers; Table S7: Secondary SCI-versus-non-CNS-trauma LASSO feature-selection results.

## Abbreviations

The following abbreviations are used in this manuscript:

2-AG	2-arachidonoylglycerol
ABHD6	Alpha/beta hydrolase domain-containing protein 6
AUC	Area under the curve
CB2	Cannabinoid receptor type 2
CNS	Central nervous system
DEG	Differentially expressed gene
DGIdb	Drug–Gene Interaction Database
ECS	Endocannabinoid system
FDR	False discovery rate
GEO	Gene Expression Omnibus
GO-BP	Gene Ontology biological process
GO-CC	Gene Ontology cellular component
GO-MF	Gene Ontology molecular function
KEGG	Kyoto Encyclopedia of Genes and Genomes
LASSO	Least absolute shrinkage and selection operator
LOOCV	Leave-one-out cross-validation
MCC	Maximal clique centrality
miRNA	MicroRNA
NCBI	National Center for Biotechnology Information
NK	Natural killer
OMIM	Online Mendelian Inheritance in Man
PPI	Protein–protein interaction
ROC	Receiver operating characteristic
SCI	Spinal cord injury
STRING	Search Tool for the Retrieval of Interacting Genes/Proteins
TF	Transcription factor
