# Supplementary material for: Cross-Species Transcriptomic Analysis Identifies an Endocannabinoid-Associated Immune Remodeling Signature and Candidate Pharmacologic Targets in Spinal Cord Injury
Source: Biomedicines. 2026 Jun 25;14(7):1446. doi: 10.3390/biomedicines14071446 (PMC13406091; doi:10.3390/biomedicines14071446)
Supplement: Supplementary file 1 [file biomedicines-14-01446-s001.zip › biomedicines-4401102-supplementary-updated.pdf]

Supplementary Materials

Cross-species transcriptomic analysis identifies an endocannabinoid-associated immune remodeling signature and candidate pharmacologic targets in spinal cord injury

Overview

This supplementary document provides additional analyses supporting the main findings of the study. The supplementary materials include machine-learning model optimization results, disease enrichment analyses, gene–disease association visualizations, and complete enrichment output tables generated from Jensen DISEASES Curated 2025, DisGeNET, and OMIM Disease databases, together with DGIdb drug–gene interaction data and ChEA 2022 transcription factor annotations. These materials are provided to improve transparency, reproducibility, and biological interpretation of the ECS–immune remodeling signature identified in spinal cord injury. In response to peer-review comments, additional sensitivity analyses comparing SCI patients with non-CNS trauma controls were performed, including differential-expression and ROC-based evaluation of ECS-associated biomarkers.

Supplementary Figures

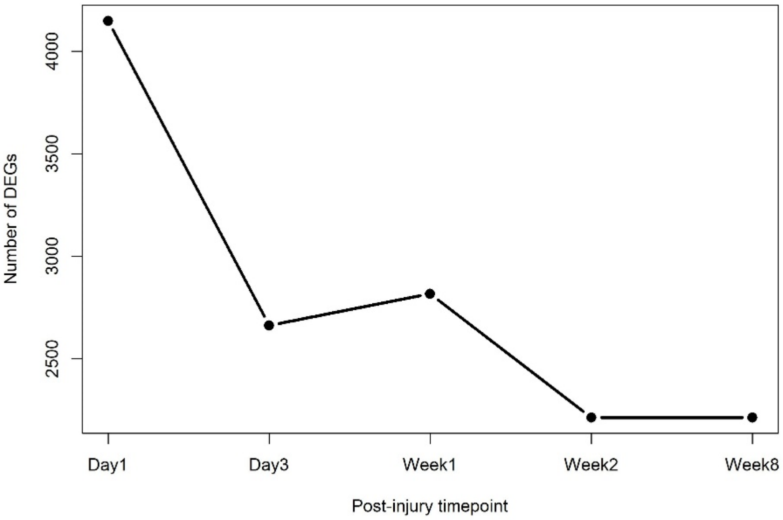

Figure S1. Temporal transcriptomic remodeling after SCI in the rat discovery cohort (GSE45006). Differentially expressed genes (DEGs) were identified at Day 1, Day 3, Week 1, Week 2, and Week 8 after SCI relative to sham controls using the limma framework (adjusted $P < 0.05$). The number of DEGs was highest at Day 1 (n = 4,148), decreased at Day 3 (n = 2,663), increased modestly at Week 1 (n = 2,817), and subsequently stabilized at Week 2 (n = 2,213) and Week 8 (n = 2,214). These findings indicate extensive early transcriptomic remodeling followed by partial stabilization during the chronic phase after SCI.

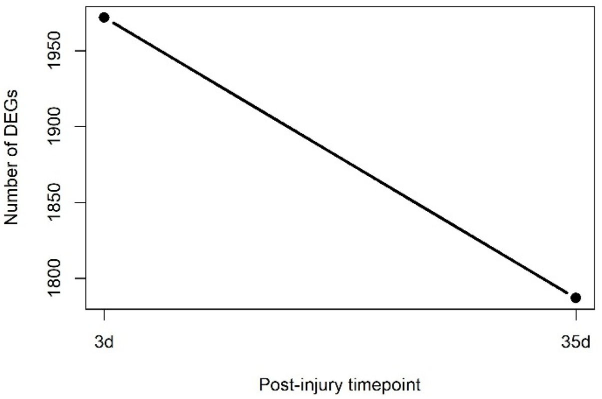

Figure S2. Temporal transcriptomic remodeling in the mouse SCI validation cohort (GSE171441). Line plot showing the total number of differentially expressed genes (DEGs) identified at 3 days and 35 days after SCI relative to time-matched sham controls. A total of 1,972 DEGs were identified at 3 days, whereas 1,787 DEGs were identified at 35 days.

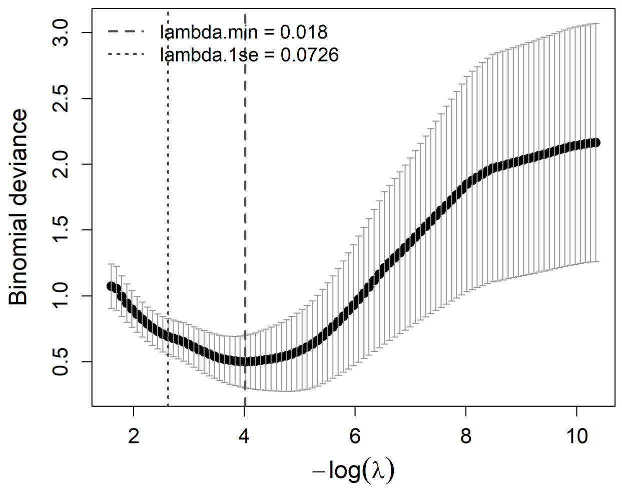

Figure S3. Leave-one-out cross-validation (LOOCV) curve used for LASSO model selection. Vertical dashed lines indicate λmin and λ1se values. The λ1se criterion was selected to obtain the most parsimonious diagnostic model.

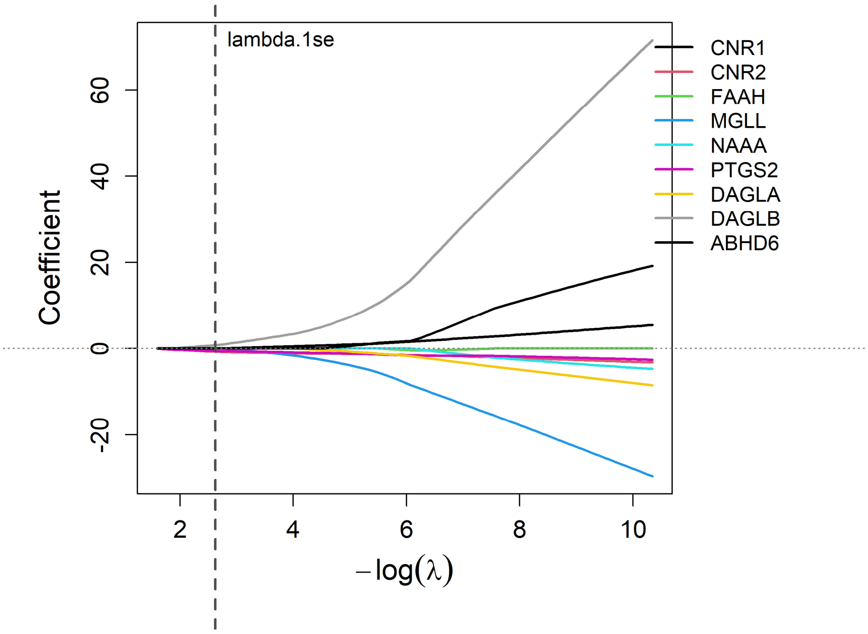

Figure S4. LASSO coefficient trajectories for ECS-related genes across the regularization path. The vertical dashed line indicates the selected λ1se value used for final model construction. At λ1se, only *CNR2*, *PTGS2*, and *DAGLB* retained non-zero coefficients in the final diagnostic signature.

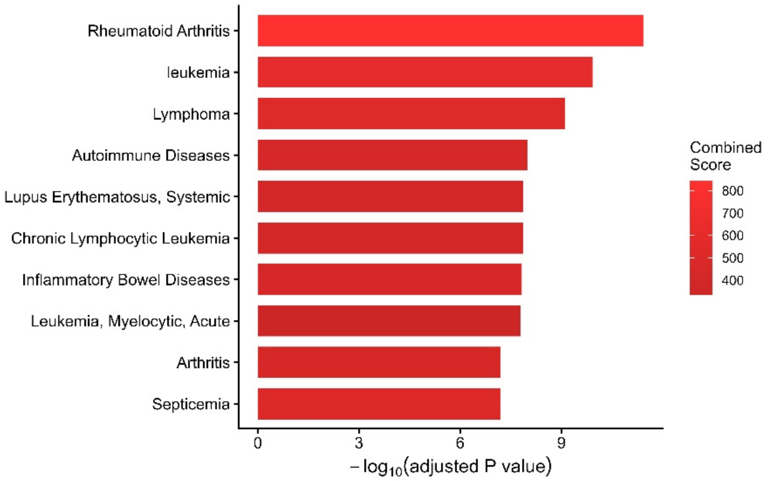

Figure S5. DisGeNET disease enrichment bar plot of the 28 ECS–immune-associated genes. The bar plot shows the top enriched disease terms obtained from the DisGeNET library in Enrichr. Enriched annotations included autoimmune, inflammatory, hematological, lymphoproliferative, infectious, and vascular disease-related terms.

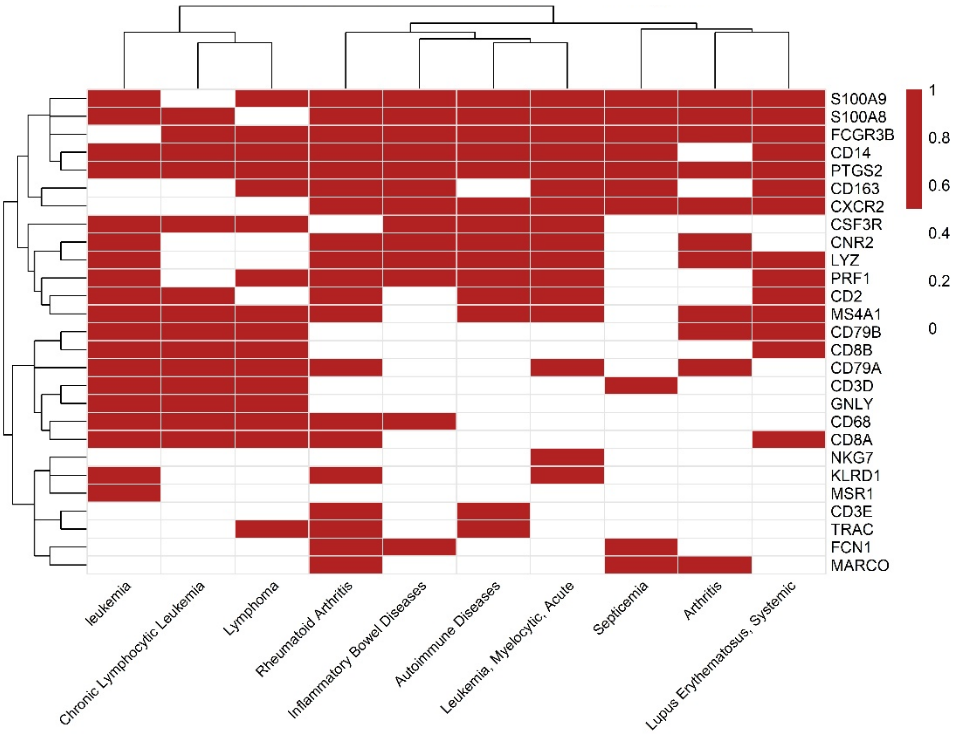

Figure S6. Gene–disease association clustergram based on DisGeNET enrichment analysis. Rows represent input genes and columns represent enriched disease terms. Colored cells indicate the presence of a gene–disease association within the DisGeNET enrichment output.

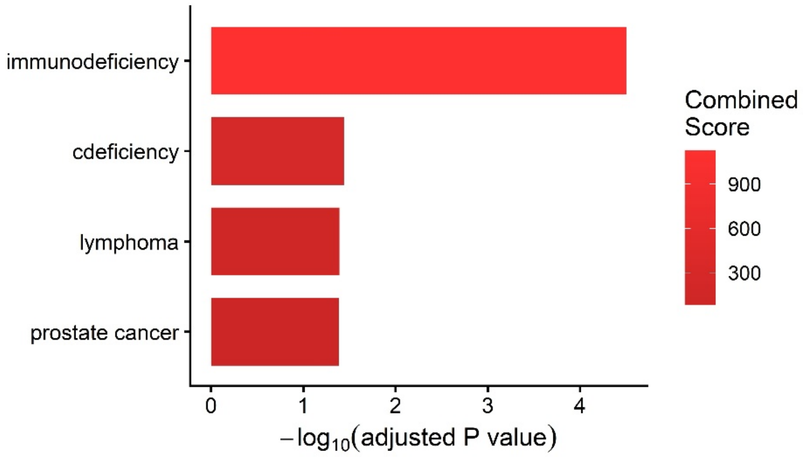

Figure S7. OMIM Disease enrichment bar plot of the 28 ECS–immune-associated genes. The bar plot shows the enriched disease terms obtained from the OMIM Disease library in Enrichr. The analysis identified a limited set of disease annotations, primarily including immunodeficiency, lymphoma, and prostate cancer-related terms.

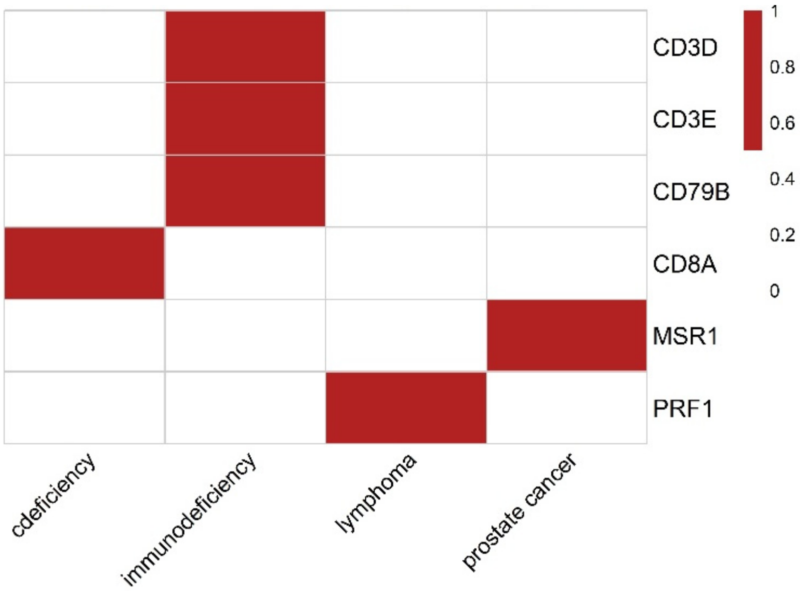

Figure S8. Gene–disease association heatmap based on OMIM Disease enrichment analysis. Rows represent input genes and columns represent enriched OMIM Disease terms. Colored cells indicate gene–disease associations identified in the OMIM Disease enrichment output.

Supplementary Tables

Table S1. Complete disease enrichment results generated using the Jensen DISEASES Curated 2025 database in Enrichr for the 28 ECS–immune-associated genes. The table corresponds to the original Enrichr output and includes all enriched disease terms together with the associated overlap statistics, p-values, adjusted p-values, odds ratios, combined scores, and contributing genes. (E-notation values, when present, are displayed as ×10^ without rounding.)

Term	Overlap	P-value	Adjusted P-value	Odds Ratio	Combined Score	Genes
IMMUNE SYSTEM DISEASE	4/243	$3.460599029938223 \times 10^{-4}$	$1.4880575828734359 \times 10^{-2}$	13.760808926080893	109.65849191961024	*CD2; PRF1; CD3D; MS4A1*
PRIMARY IMMUNODEFICIENCY DISEASE	3/216	$3.3347480488947283 \times 10^{-3}$	$7.169708305123666 \times 10^{-2}$	11.131830985915492	63.488818978235464	*CD2; CD3D; MS4A1*
SEVERE CONGENITAL NEUTROPENIA	1/9	$1.2532045363447937 \times 10^{-2}$	$8.981299177137689 \times 10^{-2}$	92.42592592592592	404.7762265532091	*CSF3R*
NEUTROPENIA	1/9	$1.2532045363447937 \times 10^{-2}$	$8.981299177137689 \times 10^{-2}$	92.42592592592592	404.7762265532091	*CSF3R*
LEUKOPENIA	1/9	$1.2532045363447937 \times 10^{-2}$	$8.981299177137689 \times 10^{-2}$	92.42592592592592	404.7762265532091	*CSF3R*
AGRANULOCYTOSIS	1/9	$1.2532045363447937 \times 10^{-2}$	$8.981299177137689 \times 10^{-2}$	92.42592592592592	404.7762265532091	*CSF3R*
COMMON VARIABLE IMMUNODEFICIENCY	1/13	$1.805312960024282 \times 10^{-2}$	$1.0161231332591465 \times 10^{-1}$	61.60493827160494	247.30909578576168	*MS4A1*
AGAMMAGLOBULINEMIA	1/16	$2.21743989287616 \times 10^{-2}$	$1.0161231332591465 \times 10^{-1}$	49.27654320987654	187.6853284304674	*MS4A1*
AUTOIMMUNE DISEASE	2/170	$2.3491133424862417 \times 10^{-2}$	$1.0161231332591465 \times 10^{-1}$	9.067765567765568	34.01438767621528	*CD2; MS4A1*
LYMPHATIC SYSTEM DISEASE	1/19	$2.627898816211653 \times 10^{-2}$	$1.0161231332591465 \times 10^{-1}$	41.05761316872428	149.40806261137496	*PRF1*
B CELL DEFICIENCY	1/19	$2.627898816211653 \times 10^{-2}$	$1.0161231332591465 \times 10^{-1}$	41.05761316872428	149.40806261137496	*MS4A1*
FAMILIAL VISCERAL AMYLOIDOSIS	1/21	$2.9006146168748567 \times 10^{-2}$	$1.0161231332591465 \times 10^{-1}$	36.94814814814815	130.8055903894323	*LYZ*
LEUKOCYTE DISEASE	1/24	$3.308307875727454 \times 10^{-2}$	$1.0161231332591465 \times 10^{-1}$	32.12399355877617	109.50212795754759	*CSF3R*
SEVERE COMBINED IMMUNODEFICIENCY	1/24	$3.308307875727454 \times 10^{-2}$	$1.0161231332591465 \times 10^{-1}$	32.12399355877617	109.50212795754759	*CD3D*
COMBINED IMMUNODEFICIENCY	1/29	$3.9841313496312473 \times 10^{-2}$	$1.1421176535609576 \times 10^{-1}$	26.38095238095238	85.02187553087816	*CD3D*
PROSTATE CANCER	1/36	$4.922636939208551 \times 10^{-2}$	$1.1759632688109316 \times 10^{-1}$	21.097354497354498	63.53100866506492	*MSR1*
PROSTATE DISEASE	1/36	$4.922636939208551 \times 10^{-2}$	$1.1759632688109316 \times 10^{-1}$	21.097354497354498	63.53100866506492	*MSR1*
MALE REPRODUCTIVE ORGAN CANCER	1/36	$4.922636939208551 \times 10^{-2}$	$1.1759632688109316 \times 10^{-1}$	21.097354497354498	63.53100866506492	*MSR1*
MALE REPRODUCTIVE SYSTEM DISEASE	1/40	$5.454947834145277 \times 10^{-2}$	$1.2345408256223521 \times 10^{-1}$	18.929724596391264	55.05988911315167	*MSR1*
REPRODUCTIVE ORGAN CANCER	1/54	$7.295498516431843 \times 10^{-2}$	$1.5685321810328462 \times 10^{-1}$	13.919636617749825	36.44039305811433	*MSR1*
AMYLOIDOSIS	1/70	$9.356685210249617 \times 10^{-2}$	$1.853697035277246 \times 10^{-1}$	10.683306494900698	25.309598161887177	*LYZ*
RHEUMATOID ARTHRITIS	1/71	$9.484031343278933 \times 10^{-2}$	$1.853697035277246 \times 10^{-1}$	10.53015873015873	24.80442820569768	*CD2*
ARTHRITIS	1/88	$1.16227015961519 \times 10^{-1}$	$2.172939863628399 \times 10^{-1}$	8.465304384844615	18.219112464159224	*CD2*
BONE INFLAMMATION DISEASE	1/99	$1.2980492369995256 \times 10^{-1}$	$2.25370319279169 \times 10^{-1}$	7.510959939531368	15.335296224041254	*CD2*
REPRODUCTIVE SYSTEM DISEASE	1/100	$1.310292553948657 \times 10^{-1}$	$2.25370319279169 \times 10^{-1}$	7.434717545828657	15.109834134055738	*MSR1*
DISEASE OF ANATOMICAL ENTITY	6/2727	$1.7337337954525905 \times 10^{-1}$	$2.845377615353162 \times 10^{-1}$	1.7290768768166784	3.029874806268542	*CD2; MSR1; CSF3R; PRF1; CD3D; MS4A1*
AUTOIMMUNE DISEASE OF MUSCULOSKELETAL SYSTEM	1/140	$1.7866324561519853 \times 10^{-1}$	$2.845377615353162 \times 10^{-1}$	5.284572342126299	9.101368215344134	*CD2*
HEMATOPOIETIC SYSTEM DISEASE	1/170	$2.1272596505799363 \times 10^{-1}$	$3.266863034819188 \times 10^{-1}$	4.339907955292571	6.717094667010364	*CSF3R*
ORGAN SYSTEM CANCER	1/204	$2.4968402587400798 \times 10^{-1}$	$3.702211418131843 \times 10^{-1}$	3.6068235723408137	5.004680714855542	*MSR1*
DISEASE	7/3737	$2.5900344145719295 \times 10^{-1}$	$3.7123826608864324 \times 10^{-1}$	1.4514745308310992	1.9608171625822695	*CD2; MSR1; CSF3R; PRF1; LYZ; CD3D; MS4A1*
BONE DISEASE	1/236	$2.8293589245138157 \times 10^{-1}$	$3.9245946372288415 \times 10^{-1}$	3.1106382978723404	3.9272895225835924	*CD2*
AUTOSOMAL GENETIC DISEASE	2/898	$3.600790677708136 \times 10^{-1}$	$4.838562473170308 \times 10^{-1}$	1.637706043956044	1.6728047685979912	*LYZ; MS4A1*
CONNECTIVE TISSUE DISEASE	1/354	$3.9369881977601756 \times 10^{-1}$	$5.130014924354168 \times 10^{-1}$	2.058440877137761	1.9188149362301623	*CD2*

PHYSICAL DISORDER	1/438	$4.622903685593569 \times 10^{-1}$	$5.538902398232093 \times 10^{-1}$	1.655648783795237	1.2774358226041904	*CSF3R*
AUTOSOMAL DOMINANT DISEASE	1/444	$4.6689234477945457 \times 10^{-1}$	$5.538902398232093 \times 10^{-1}$	1.632723016470195	1.2435742173596644	*LYZ*
CANCER	1/484	$4.966157587278212 \times 10^{-1}$	$5.538902398232093 \times 10^{-1}$	1.4944406103826393	1.0460167779624006	*MSR1*
DISEASE OF CELLULAR PROLIFERATION	1/487	$4.9877940422442035 \times 10^{-1}$	$5.538902398232093 \times 10^{-1}$	1.4849870446578266	1.032944153041981	*MSR1*
MONOGENIC DISEASE	2/1187	$5.01321289962585 \times 10^{-1}$	$5.538902398232093 \times 10^{-1}$	1.219539110678351	0.84210161725083	*LYZ; MS4A1*
AUTOSOMAL RECESSIVE DISEASE	1/492	$5.023655663512828 \times 10^{-1}$	$5.538902398232093 \times 10^{-1}$	1.4694878177566568	1.0116353904111122	*MS4A1*
MUSCULOSKELETAL SYSTEM DISEASE	1/584	$5.640997524904295 \times 10^{-1}$	$5.968654371692179 \times 10^{-1}$	1.231751477034496	0.7052075006641921	*CD2*
INHERITED METABOLIC DISORDER	1/592	$5.691042540450683 \times 10^{-1}$	$5.968654371692179 \times 10^{-1}$	1.2145766748135614	0.6846467157411258	*LYZ*
DISEASE OF METABOLISM	1/670	$6.150820507412783 \times 10^{-1}$	$6.297268614732134 \times 10^{-1}$	1.068648618723357	0.5193628057660915	*LYZ*
GENETIC DISEASE	2/1648	$6.838697128882867 \times 10^{-1}$	$6.838697128882867 \times 10^{-1}$	0.8564351808580242	0.32543496966429974	*LYZ; MS4A1*

Table S2A. Complete disease enrichment results generated using the DisGeNET database in Enrichr for the 28 ECS–immune-associated genes.

Term	Overlap	P-value	Adjusted P-value	Odds Ratio	Combined Score
Rheumatoid Arthritis	20/1833	$2.398987428422397 \times 10^{-15}$	$3.7975970991926546 \times 10^{-12}$	25.039988968560397	842.9394191118477
Autoimmune Diseases	14/1060	$2.5320816537110834 \times 10^{-11}$	$1.0020713144561613 \times 10^{-8}$	18.093690248565967	441.4750821825463
Lupus Erythematosus, Systemic	14/1113	$4.854341167373354 \times 10^{-11}$	$1.3922448660038484 \times 10^{-8}$	17.172884440400363	407.83132170974426
Inflammatory Bowel Diseases	13/911	$6.617288699733478 \times 10^{-11}$	$1.4964525730968707 \times 10^{-8}$	18.40846325167038	431.47137354150976
Ulcerative Colitis	9/915	$2.654899120562196 \times 10^{-6}$	$7.782787607129549 \times 10^{-5}$	9.968281631230393	127.98380356796818
Crohn Disease	8/912	$2.493609544631477 \times 10^{-5}$	$4.6992665585138434 \times 10^{-4}$	8.43716814159292	89.42718352832861
Celiac Disease	5/312	$6.548549917972089 \times 10^{-5}$	$9.779579735990393 \times 10^{-4}$	13.925081433224756	134.149803934423
Multiple Sclerosis	8/1106	$9.812795263182513 \times 10^{-5}$	$1.3164114323405015 \times 10^{-3}$	6.875774134790528	63.458157930569854
Psoriasis	9/818	$1.0500031746253716 \times 10^{-6}$	$4.2619359626460596 \times 10^{-5}$	11.220284952182682	154.46649175145276
Arthritis, Psoriatic	4/201	$1.6779013858108356 \times 10^{-4}$	$1.9247231114047484 \times 10^{-3}$	16.730118443316414	145.4315156265583
Vasculitis	4/136	$3.689487722451495 \times 10^{-5}$	$6.562313555776087 \times 10^{-4}$	25.050505050505052	255.70147329287963
Systemic Scleroderma	6/562	$1.0657139135219314 \times 10^{-4}$	$1.3942356405828244 \times 10^{-3}$	9.523871811641596	87.11195502362146
Sarcoidosis	7/236	$2.7936463749900296 \times 10^{-8}$	$2.4568567842273425 \times 10^{-6}$	28.737991266375545	499.8494534848449

Graft-vs-Host Disease	8/226	$6.007417295347831 \times 10^{-10}$	$8.645219616850559 \times 10^{-8}$	36.24587155963303	769.603371721271
Immunologic Deficiency Syndromes	8/641	$1.876989151106959 \times 10^{-6}$	$6.0638241351067676 \times 10^{-5}$	12.220537124802528	161.13806655367014
Autoimmune Lymphoproliferative Syndrome	2/31	$8.570688316410276 \times 10^{-4}$	$6.195159636930351 \times 10^{-3}$	52.89920424403183	373.5737744029888
Post-transplant lymphoproliferative disorder	2/17	$2.537242832581918 \times 10^{-4}$	$2.625134250965475 \times 10^{-3}$	102.34358974358975	847.3294323481033
Immune thrombocytopenic purpura	5/183	$5.030271055540114 \times 10^{-6}$	$1.263955409669841 \times 10^{-4}$	24.174401563263313	294.9285859707303
Lymphohistiocytosis, Hemophagocytic	3/51	$4.892036785487742 \times 10^{-5}$	$7.902136970843975 \times 10^{-4}$	49.81	494.3800262044663
IGA Glomerulonephritis	8/237	$8.7514665301215 \times 10^{-10}$	$1.0656593474755642 \times 10^{-7}$	34.48558951965065	719.2531685343632
Glomerulonephritis	4/210	$1.9845722140515243 \times 10^{-4}$	$2.15176562660518 \times 10^{-3}$	15.991909385113269	136.33001986358033
Leukemia, Myelocytic, Acute	16/1703	$8.074971349987848 \times 10^{-11}$	$1.5978349558788454 \times 10^{-8}$	14.451689389448726	335.85244468903426
Chronic Lymphocytic Leukemia	14/1120	$5.276986226167461 \times 10^{-11}$	$1.3922448660038484 \times 10^{-8}$	17.057866184448464	403.6757828726365
MYELODYSPLASTIC SYNDROME	8/683	$3.0099261322761866 \times 10^{-6}$	$8.663114667987642 \times 10^{-5}$	11.43525925925926	145.383255174091
Multiple Myeloma	11/1312	$7.049299293908476 \times 10^{-7}$	$3.20475440535149 \times 10^{-5}$	9.286114753357147	131.5393702555781
Lymphoma, Non-Hodgkin	7/539	$7.187961296108759 \times 10^{-6}$	$1.6580180128336502 \times 10^{-4}$	12.180451127819548	144.25433697132934
Mucosa-Associated Lymphoid Tissue Lymphoma	6/176	$1.3677290391855053 \times 10^{-7}$	$9.516121630722607 \times 10^{-6}$	31.767914438502675	502.09010622411137
Mucocutaneous Lymph Node Syndrome	5/156	$2.301684017298724 \times 10^{-6}$	$7.103592962598661 \times 10^{-5}$	28.535847970054707	370.44865503286775

Table S2B. Contributing genes for DisGeNET disease enrichment terms.

Term	Contributing genes
Rheumatoid Arthritis	*FCN1; CD163; TRAC; PRF1; PTGS2; CD3E; LYZ; CD2; CD79A; MARCO; FCGR3B; CNR2; CD8A; CXCR2; KLRD1; CD14; CD68; S100A9; MS4A1; S100A8*
Autoimmune Diseases	*CSF3R; TRAC; PRF1; PTGS2; CD3E; LYZ; CD2; FCGR3B; CNR2; CXCR2; CD14; S100A9; MS4A1; S100A8*
Lupus Erythematosus, Systemic	*CD163; PRF1; PTGS2; LYZ; CD79B; CD2; FCGR3B; CD8B; CD8A; CXCR2; CD14; S100A9; MS4A1; S100A8*
Inflammatory Bowel Diseases	*FCN1; CD163; CSF3R; PRF1; PTGS2; LYZ; FCGR3B; CNR2; CXCR2; CD14; CD68; S100A9; S100A8*
Ulcerative Colitis	*CD163; FCGR3B; CXCR2; CD14; PTGS2; LYZ; CD68; S100A9; S100A8*
Crohn Disease	*CD163; FCGR3B; CD14; PTGS2; LYZ; CD3D; S100A9; MS4A1*
Celiac Disease	*CNR2; PRF1; CD14; CD3E; LYZ*
Multiple Sclerosis	*FCGR3B; CNR2; CD8A; CXCR2; PRF1; CD14; CD68; MS4A1*
Psoriasis	*CD163; FCGR3B; CXCR2; PRF1; CD14; LYZ; CD68; S100A9; S100A8*
Arthritis, Psoriatic	*PTGS2; CD68; S100A9; S100A8*
Vasculitis	*FCGR3B; S100A9; MS4A1; S100A8*
Systemic Scleroderma	*CNR2; CXCR2; CD14; PTGS2; CD68; MS4A1*
Sarcoidosis	*CD163; FCGR3B; CD14; PTGS2; CD68; S100A9; S100A8*
Graft-vs-Host Disease	*FCGR3B; GNLY; PRF1; CD14; CD3E; S100A9; MS4A1; S100A8*
Immunologic Deficiency Syndromes	*CD79B; CD79A; PRF1; PTGS2; CD3E; CD68; CD3D; MS4A1*
Autoimmune Lymphoproliferative Syndrome	*PRF1; MS4A1*
Post-transplant lymphoproliferative disorder	*FCGR3B; MS4A1*
Immune thrombocytopenic purpura	*FCGR3B; CNR2; PRF1; MS4A1; S100A8*
Lymphohistiocytosis, Hemophagocytic	*CD163; GNLY; PRF1*
IGA Glomerulonephritis	*CD79A; CD163; FCGR3B; TRAC; PRF1; CD14; CD68; MS4A1*
Glomerulonephritis	*CD163; FCGR3B; PRF1; MS4A1*

Leukemia, Myelocytic, Acute	*CD163; CSF3R; PRF1; NKG7; PTGS2; LYZ; CD2; CD79A; FCGR3B; CNR2; CXCR2; KLRD1; CD14; S100A9; MS4A1; S100A8*
Chronic Lymphocytic Leukemia	*CSF3R; PTGS2; CD3D; CD79B; CD2; CD79A; FCGR3B; CD8B; GNLY; CD8A; CD14; CD68; MS4A1; S100A8*
MYELODYSPLASTIC SYNDROME	*CSF3R; FCGR3B; CXCR2; CD14; CD68; S100A9; MS4A1; S100A8*
Multiple Myeloma	*CD79B; CD79A; FCGR3B; CNR2; GNLY; PRF1; CD14; PTGS2; CD68; S100A9; MS4A1*
Lymphoma, Non-Hodgkin	*CD79B; CD79A; FCGR3B; PRF1; CD14; PTGS2; MS4A1*
Mucosa-Associated Lymphoid Tissue Lymphoma	*CD79B; CD79A; CXCR2; CD14; PTGS2; S100A8*
Mucocutaneous Lymph Node Syndrome	*FCGR3B; PRF1; CD14; S100A9; S100A8*

Table S3. Complete disease enrichment results generated using the OMIM Disease database in Enrichr for the 28 ECS–immune-associated genes. The table corresponds to the original Enrichr output and includes all enriched disease terms together with the associated overlap statistics, p-values, adjusted p-values, odds ratios, combined scores, and contributing genes.

Term	Overlap	P-value	Adjusted P-value	Odds Ratio	Combined Score	Genes
immunodeficiency	3/28	$7.863267097660281 \times 10^{-6}$	$3.1453068390641124 \times 10^{-5}$	95.7456	1125.32756250321	*CD79B; CD3E; CD3D*
CD8A deficiency	1/13	$1.805312960024282 \times 10^{-2}$	$3.610625920048564 \times 10^{-2}$	61.6049382716 0494	247.30909578576168	*CD8A*
lymphoma	1/22	$3.03669619032 49945 \times 10^{-2}$	$4.048928253766659 \times 10^{-2}$	35.18694885361552	122.95727551819456	*PRF1*
prostate cancer	1/30	$4.118748390134586 \times 10^{-2}$	$4.118748390134586 \times 10^{-2}$	25.46998722860792	81.2396025067177	*MSR1*

Table S4. Complete list of approved drug–gene interactions identified by DGIdb analysis. Approved drug–gene interactions involving ECS-related and immune-associated genes were retrieved from DGIdb. Interaction scores are reported as provided by the DGIdb platform; drug indication information was omitted to improve table readability.

**Gene**	**Drug**	**Regulatory Approval**	**Interaction Score**
CXCR2	BEVACIZUMAB	Approved	0.14501055

CXCR2	CLOTRIMAZOLE	Approved	0.07838408
CXCR2	GENISTEIN	Approved	0.10000728
CXCR2	ACETYLCYSTEINE	Approved	0.38669480
CXCR2	MECHLORETHAMINE	Approved	0.52731110
CXCR2	IBUPROFEN, SODIUM SALT	Approved	0.07436439
CXCR2	CYCLOPHOSPHAMIDE ANHYDROUS	Approved	0.07161015
CD3E	MUROMONAB-CD3	Approved	0.82863172
CD3E	TEPLIZUMAB	Approved	1.45010552
CD3E	IPRAGLIFLOZIN	Approved	0.36252638
CD3E	DAPAGLIFLOZIN PROPANEDIOL	Approved	0.36252638
CD3E	MOSUNETUZUMAB	Approved	0.36252638
CD3E	BEXAGLIFLOZIN	Approved	0.48336851
CD3E	EPCORITAMAB	Approved	0.36252638
CD3E	GLOFITAMAB	Approved	0.36252638
CD3E	SOTAGLIFLOZIN	Approved	0.29002110
CD3E	ELRANATAMAB	Approved	0.36252638
CD3E	EMPAGLIFLOZIN	Approved	0.36252638
CD3E	CATUMAXOMAB	Approved	0.58004221
CD3E	ERTUGLIFLOZIN	Approved	0.36252638
CD3E	TALQUETAMAB	Approved	0.36252638
CD3E	TECLISTAMAB	Approved	0.36252638
CD3E	BLINATUMOMAB	Approved	0.29002110
CD3E	CANAGLIFLOZIN	Approved	0.36252638
PTGS2	CARPROFEN	Approved	0.89237262
PTGS2	INDOMETHACIN SODIUM	Approved	0.22309316
PTGS2	MEFENAMIC ACID	Approved	0.12748180
PTGS2	MECLOFENAMATE SODIUM	Approved	0.12748180

PTGS2	DICLOFENAC SODIUM	Approved	0.06561563
PTGS2	CAPECITABINE	Approved	0.01749750
PTGS2	PIRPROFEN	Approved	0.22309316
PTGS2	ESFLURBIPROFEN	Approved	0.11154658
PTGS2	SULFASALAZINE	Approved	0.03718219
PTGS2	BROMFENAC	Approved	0.29745754
PTGS2	IBUPROFEN, SODIUM SALT	Approved	0.04576270
PTGS2	ALOXIPRIN	Approved	0.22309316
PTGS2	FLURBIPROFEN	Approved	0.27886645
PTGS2	SALICYLIC ACID	Approved	0.29745754
PTGS2	ATENOLOL	Approved	0.02478813
PTGS2	DEXKETOPROFEN	Approved	0.11154658
PTGS2	ETORICOXIB	Approved	1.33855894
PTGS2	LUMIRACOXIB	Approved	0.44618631
PTGS2	SUPROFEN	Approved	0.33463973
PTGS2	NABUMETONE	Approved	0.22309316
PTGS2	IBUPROFEN LYSINE	Approved	0.22309316
PTGS2	ACECLOFENAC	Approved	0.14872877
PTGS2	IBUFENAC	Approved	0.14872877
PTGS2	BALSALAZIDE	Approved	0.11154658
PTGS2	DIFLUNISAL	Approved	0.35694905
PTGS2	BALSALAZIDE DISODIUM	Approved	0.11154658
PTGS2	KETOROLAC TROMETHAMINE	Approved	0.16731987
PTGS2	BENZYDAMINE HYDROCHLORIDE	Approved	0.22309316
PTGS2	RALOXIFENE HYDROCHLORIDE	Approved	0.01784745
PTGS2	ETODOLAC	Approved	0.66927947
PTGS2	ROFECOXIB	Approved	0.29745754

PTGS2	OXAPROZIN POTASSIUM	Approved	0.22309316
PTGS2	LORNOXICAM	Approved	0.08923726
PTGS2	TOLMETIN	Approved	0.44618631
PTGS2	FLURBIPROFEN SODIUM	Approved	0.22309316
PTGS2	MELOXICAM	Approved	0.33463973
PTGS2	SALSALATE	Approved	0.44618631
PTGS2	INDOMETHACIN	Approved	0.10039192
PTGS2	DICLOFENAC EPOLAMINE	Approved	0.22309316
PTGS2	RESERPINE	Approved	0.03432202
PTGS2	AMINOSALICYLIC ACID	Approved	0.22309316
PTGS2	TOLMETIN SODIUM	Approved	0.14872877
PTGS2	ROBENACOXIB	Approved	0.44618631
PTGS2	APAZONE	Approved	0.14872877
PTGS2	FENCLOFENAC	Approved	0.22309316
PTGS2	BISMUTH SUBSALICYLATE	Approved	0.11154658
PTGS2	CURCUMIN	Approved	0.02028120
PTGS2	THALIDOMIDE	Approved	0.01205909
PTGS2	OXAPROZIN	Approved	0.22309316
PTGS2	KETOPROFEN	Approved	0.25496361
PTGS2	INDOPROFEN	Approved	0.03718219
PTGS2	NEPAFENAC	Approved	0.44618631
PTGS2	PIROXICAM-BETA-CYCLODEXTRIN	Approved	0.06864405
PTGS2	GLAFENINE	Approved	0.06374090
PTGS2	DICLOFENAC POTASSIUM	Approved	0.22309316
PTGS2	DIPYRONE	Approved	0.08923726
PTGS2	OMEGA-3 FATTY ACIDS	Approved	0.89237262
PTGS2	ISOXICAM	Approved	0.22309316
PTGS2	OLSALAZINE SODIUM	Approved	0.11154658

PTGS2	BENZYDAMINE	Approved	0.11154658
PTGS2	MECLOFENAMATE	Approved	0.12748180
PTGS2	OXALIPLATIN	Approved	0.02348349
PTGS2	OXYPHENBUTAZONE	Approved	0.03432202
PTGS2	HYDROXYCHLOROQUINE	Approved	0.04957626
PTGS2	SULINDAC	Approved	0.06374090
PTGS2	ACEMETACIN	Approved	0.22309316
PTGS2	CYCLOSPORINE	Approved	0.01025716
PTGS2	PHENYLBUTAZONE	Approved	0.12748180
PTGS2	CELECOXIB	Approved	0.02663799
PTGS2	NAPROXEN SODIUM	Approved	0.15747752
PTGS2	ACETAMINOPHEN	Approved	0.02574152
PTGS2	DEXIBUPROFEN	Approved	0.33463973
PTGS2	CLOMETACIN	Approved	0.22309316
PTGS2	LOXOPROFEN	Approved	0.22309316
PTGS2	PARECOXIB SODIUM	Approved	0.44618631
PTGS2	MESALAMINE	Approved	0.06084359
PTGS2	FENOPROFEN	Approved	0.17847452
PTGS2	VALDECOXIB	Approved	0.89237262
PTGS2	PARECOXIB	Approved	0.44618631
PTGS2	FENOPROFEN CALCIUM	Approved	0.22309316
PTGS2	BROMFENAC SODIUM	Approved	0.22309316
PTGS2	ASPIRIN	Approved	0.02005332
PRF1	ALDESLEUKIN	Approved	1.16008441
PRF1	EMAPALUMAB	Approved	4.35031655
CSF3R	TBO-FILGRASTIM	Approved	3.86694804
CSF3R	TRAMETINIB DIMETHYL SULFOXIDE	Approved	0.12341324
CSF3R	PEGFILGRASTIM	Approved	3.86694804

CSF3R	LENOGRASTIM	Approved	0.17577037
CSF3R	RUXOLITINIB	Approved	1.81263189
CSF3R	TOFACITINIB	Approved	0.27621057
CSF3R	BENEGRASTIM	Approved	1.93347402
CSF3R	MOMELOTINIB	Approved	0.29745754
CSF3R	IMATINIB	Approved	0.05370761
CSF3R	IBRUTINIB	Approved	0.11373377
CSF3R	LIPEGFILGRASTIM	Approved	1.93347402
CSF3R	DASATINIB ANHYDROUS	Approved	0.10176179
CSF3R	PEXIDARTINIB	Approved	0.13810529
CSF3R	EFLAPEGRASTIM	Approved	0.96673701
CD14	FLUTICASONE	Approved	0.49955788
CD14	TIAGABINE HYDROCHLORIDE	Approved	1.58193329
CD14	LOVASTATIN	Approved	0.24337435
CD3D	EPCORITAMAB	Approved	0.40784218
CD3D	CANAGLIFLOZIN	Approved	0.40784218
CD3D	MOSUNETUZUMAB	Approved	0.40784218
CD3D	MUROMONAB-CD3	Approved	0.46610534
CD3D	CATUMAXOMAB	Approved	0.65254748
CD3D	BLINATUMOMAB	Approved	0.32627374
CD3D	TECLISTAMAB	Approved	0.40784218
CD3D	BEXAGLIFLOZIN	Approved	0.54378957
CD3D	ERTUGLIFLOZIN	Approved	0.40784218
CD3D	SOTAGLIFLOZIN	Approved	0.32627374
CD3D	GLOFITAMAB	Approved	0.40784218
CD3D	EMPAGLIFLOZIN	Approved	0.40784218
CD3D	ELRANATAMAB	Approved	0.40784218
CD3D	TALQUETAMAB	Approved	0.40784218

CD3D	IPRAGLIFLOZIN	Approved	0.40784218
CD3D	DAPAGLIFLOZIN PROPANEDIOL	Approved	0.40784218
CD68	FUROSEMIDE	Approved	0.96673701
CD68	BUMETANIDE	Approved	0.96673701
CNR2	NABILONE	Approved	1.50587880
CNR2	DRONABINOL	Approved	0.11810814
CD163	FLUTICASONE	Approved	2.74756835
CD79B	POLATUZUMAB VEDOTIN	Approved	1.37378417

Table S5. Detailed ChEA 2022 transcription factor annotations and overlapping target genes.

TF	Full ChEA 2022 term	Overlapping target genes
E2A	27217539 ChIP-Seq RAMOS-Cell Line Human	*FCN1, CD79B, CNR2, PRF1, KLRD1, DAGLB, CD3E, MS4A1*
GATA3	27048872 ChIP-Seq THYMUS Human	*CD2, CSF3R, CD8B, CD8A, GNLY, KLRD1, CD3E, CD3D*
LYL1	30185409 ChIP-Seq K562 Mouse Bone Marrow Leukemia	*CD2, CSF3R, PRF1, CD14, DAGLB, PTGS2*
SPI1	23547873 ChIP-Seq NB4 Human	*FCN1, CD163, CNR2, CXCR2, NKG7, CD14, LYZ, CD68, S100A9, S100A8*
FLI1	20887958 ChIP-Seq HPC-7 Mouse	*CSF3R, CNR2, CXCR2, PRF1, NKG7, KLRD1, PTGS2*
GATA1	30185409 ChIP-Seq K562 Mouse Bone Marrow Leukemia	*CD2, CSF3R, PRF1, CD14, DAGLB*
E2F1	20622854 ChIP-Seq HELA Human	*CD2, FCN1, CD8A, CD14, DAGLB, CD3E, S100A8*
GATA6	21074721 ChIP-Seq CACO-2 Mouse	*CD2, CSF3R, CNR2, PRF1, PTGS2, S100A9, S100A8*
CUX1	19635798 ChIP-ChIP Multiple Cancer Types Human	*CD163, CD14, LYZ, CD68, CD3E, MS4A1, CD3D*
TAL1	30185409 ChIP-Seq K562 Mouse Bone Marrow Leukemia	*CD2, CSF3R, PRF1, CD14, DAGLB*

Table S6. Secondary SCI-versus-non-CNS-trauma sensitivity analysis of ECS-associated biomarkers. Differential-expression statistics were obtained from the SCI versus non-CNS trauma comparison in GSE151371. ROC-based AUC values represent individual-gene discriminatory performance for SCI versus non-CNS trauma controls.

Gene	logFC	P value	Adjusted P value	AUC
CNR2	-0.918	0.0146	0.0648	0.753
PTGS2	-1.223	0.00049	0.0087	0.847
DAGLB	0.143	0.292	0.458	0.647

The combined CNR2–PTGS2–DAGLB logistic regression model achieved an AUC of 0.900 for discriminating SCI patients from non-CNS trauma controls.

Table S7. Secondary SCI-versus-non-CNS-trauma LASSO feature-selection results.

Gene	λ1se coefficient
CNR2	-0.121
PTGS2	-0.489
DAGLB	0

The LASSO-derived two-gene signature achieved an AUC of 0.868 in the SCI-versus-non-CNS-trauma comparison.
